# Artificial intelligence reveals features associated with breast cancer neoadjuvant chemotherapy responses from multi-stain histopathologic images

**DOI:** 10.1038/s41698-023-00352-5

**Published:** 2023-01-27

**Authors:** Zhi Huang, Wei Shao, Zhi Han, Ahmad Mahmoud Alkashash, Carlo De la Sancha, Anil V. Parwani, Hiroaki Nitta, Yanjun Hou, Tongxin Wang, Paul Salama, Maher Rizkalla, Jie Zhang, Kun Huang, Zaibo Li

**Affiliations:** 1 School of Electrical and Computer Engineering, Purdue University, West Lafayette, IN 47907 USA; 2 Department of Electrical and Computer Engineering, Indiana University – Purdue University Indianapolis, Indianapolis, IN 46202 USA; 3 Department of Medicine, Indiana University School of Medicine, Indianapolis, IN 46202 USA; 4 Regenstrief Institute, Indianapolis, IN 46202 USA; 5 Department of Biostatistics and Health Data Science, Indiana University School of Medicine, Indianapolis, IN 46202 USA; 6 Department of Pathology, Indiana University School of Medicine, Indianapolis, IN 46202 USA; 7 Department of Pathology, The Ohio State University Wexner Medical Center, Columbus, OH 43210 USA; 8 Roche Tissue Diagnostics, 1910 E. Innovation Park Drive, Tucson, AZ 85755 USA; 9 University Hospitals Cleveland Medical Center, Case Western Reserve University, 11100 Euclid Avenue, Cleveland, OH 44106 USA; 10 Department of Computer Science, Indiana University Bloomington, Bloomington, IN 47408 USA; 11 Department of Medical and Molecular Genetics, Indiana University School of Medicine, Indianapolis, IN 46202 USA

**Keywords:** Breast cancer, Outcomes research, Predictive markers

## Abstract

Advances in computational algorithms and tools have made the prediction of cancer patient outcomes using computational pathology feasible. However, predicting clinical outcomes from pre-treatment histopathologic images remains a challenging task, limited by the poor understanding of tumor immune micro-environments. In this study, an automatic, accurate, comprehensive, interpretable, and reproducible whole slide image (WSI) feature extraction pipeline known as, IMage-based Pathological REgistration and Segmentation Statistics (IMPRESS), is described. We used both H&E and multiplex IHC (PD-L1, CD8+, and CD163+) images, investigated whether artificial intelligence (AI)-based algorithms using automatic feature extraction methods can predict neoadjuvant chemotherapy (NAC) outcomes in HER2-positive (HER2+) and triple-negative breast cancer (TNBC) patients. Features are derived from tumor immune micro-environment and clinical data and used to train machine learning models to accurately predict the response to NAC in breast cancer patients (HER2+ AUC = 0.8975; TNBC AUC = 0.7674). The results demonstrate that this method outperforms the results trained from features that were manually generated by pathologists. The developed image features and algorithms were further externally validated by independent cohorts, yielding encouraging results, especially for the HER2+ subtype.

## Introduction

Predicting patient outcomes based on features or grades derived from tumor histopathologic images can help understand the potential hazard factors and improve treatment planning towards precision oncology^1^. In contrast to traditional image-based quantitative analysis, artificial intelligence (AI)-based computational pathology utilizes multiple sources of histopathologic images and automatic feature calculation approaches to extract patterns and analyze features^2^. One of the objectives of such AI-based computational pathology approaches is to predict the treatment outcomes including overall survival. This has been recently demonstrated by “so called” end-to-end deep learning approaches^3,4^ and interpretable machine learning approaches backed with morphologic feature extraction^5–7^. These studies facilitated the applications of computational pathology for clinical diagnosis and prognosis, as well as the interpretation of the roles of different cellular components in the tumor immune micro-environment such as tumor-infiltrating lymphocytes (TILs), which have been discovered to play important roles in clinical outcomes of cancers^8^.

Pathologic complete response (pCR) is a presumptive surrogate for disease-free survival in breast cancer patients who have received neoadjuvant chemotherapy (NAC)^9,10^. Predicting the pCR in breast cancer patients based on pre-treatment biopsies brings tremendous clinical and treatment impact. While the image-based prediction for NAC treatment response in breast cancer patients has been explored in both the areas of radiology^11–14^ and pathology^15–18^, it is especially challenging when using pre-NAC images than post-NAC images^14^. For example, when Qu et al.^14^ adopted a deep learning approach to predict breast cancer pCR via MRI images, they observed an inferior AUC (0.553) using pre-NAC data than 0.968 using post-NAC data.

Meanwhile, the association between pCR and tumor immune micro-environment has been frequently but not systematically studied in breast cancer. For example, higher pCR rates were found in hormone receptor (HR)-negative tumors in multiple trials^19–21^, and a high Ki-67 index (≥50%) was observed to be an independent predictive factor for pCR in HER2-positive breast cancer patients^22,23^. One recent study found that PD-L1 expression was correlated with TILs and was a significant factor in predicting pCR^24^. In addition to these univariate marker analyses, cellular components of tumor immune micro-environment such as TILs are also associated with response to NAC in breast cancer^17,18,25–27^. For example, a positive association between TILs and pCR was confirmed^17,25,28–35^. Hwang et al.^16^ reported that high pre-NAC TILs is a strong prognostic marker for pCR. In ref. ^16^, pre-NAC TILs were calculated from the percentage of all mononuclear cells (including lymphocytes and plasma cells) in stromal areas, and were scored as a categorical variable in 10% increments^36^. Ali et al.^15^ extracted lymphocyte density from pre-treatment biopsies and confirmed it is one of the strongest predictors in logistic regression classifier. While Denkert et al.^17^ showed that the percentage of intratumoral lymphocytes (iTu-Ly) was the most significant independent parameter for pCR in breast cancer NAC rather than the percentage of stromal lymphocytes (str-Ly), Zhang et al.^37^ evaluated the lymphocyte-to-monocyte ratio in pre-NAC to predict pCR. Among most of these studies, TILs and other histopathologic features were evaluated manually. Meanwhile, the accuracy of NAC response prediction by machine learning algorithm and its comparison with human assessments are usually not reported. In addition, image-based statistical features are not exploited completely via multiplexed histopathologic images. To systematically study how TILs and other image-based features in pre-NAC images can predict and explain pCR outcomes from both the tumor immune micro-environment and the immunohistochemistry response, it is both research and clinical impactful to build an automatic and reproducible image-based feature extraction procedure thus systematically evaluate the association between tumor immune micro-environment and pCR.

In this study, we leveraged the multi-stain histopathologic images, proposed an automatic workflow for breast cancer pCR prediction from pre-NAC biopsies. Multiplexed histopathologic images can identify multiple markers simultaneously from a single tissue section^38^. With our approach, IHC-stained information including PD-L1, CD8+T cells, and CD163+ macrophages were co-registered into H&E-stained tumor immune micro-environment, generated a combined feature set to predict the NAC response. With all that mentioned, an automatic whole slide image (WSI) feature extraction pipeline was constructed. By taking the advantage of multiplexed histopathologic images, we extracted 36 interpretable and meaningful histopathological features, established three categories of quantitative features to characterize different cellular components – namely the “area ratio”, “proportion”, and “purity” – in our proposed pipeline, and formally designated our pipeline as “IMage-based Pathological REgistration and Segmentation Statistics”, or “IMPRESS” in short. Sixty-two HER2+ and sixty-four TNBC female patients were included in our cohort to examine whether a machine learning model using IMPRESS would be able to predict pCR for NAC. We found that the developed machine learning models utilized IMPRESS and clinical features can accurately predict the response to NAC in breast cancer patients (HER2+ AUC = 0.8975; TNBC AUC = 0.7674), and outperformed the results learned by features which were manually generated by pathologists. The developed approach was further externally validated in two independent cohorts for HER2+ and TNBC subtypes, yielding AUC = 0.90 for HER2+, and AUC = 0.59 for TNBC. These results suggest pre-NAC IMPRESS features and model can help predict post-NAC outcomes, especially for HER2+ subtype. We also compared the prediction accuracy between the model learned from IMPRESS and the model learned from features which were manually generated by pathologists. Additionally, we comprehensively evaluated those automatically extracted features by feature importance analysis, residual cancer burden analysis, and correlation analysis. These results present promising insight into the tumor immune micro-environment of breast cancer NAC patients, prompting the need for multi-stained computational analysis before the NAC treatment.

## Results

### Clinical and histopathological characteristics of the cohorts

Sixty-two HER2-positive (HER2+) BC and sixty-four TNBC female patients treated with NAC and surgical excision were included. HER2+ BC patients were treated with doxorubicin/cyclophosphamide/taxol together with anti-HER2 targeted therapy, including 24 patients (39%) with residual tumors and the other 36 patients (61%) with pCR. TNBC patients were treated with standard NAC (doxorubicin/cyclophosphamide/taxol) including 37 patients (58%) with residual tumors and the other 27 patients (32%) with pCR. The clinical and histopathologic characteristics of these patients were summarized in Table 1. In addition, the external cohort characteristics for HER2+ and TNBC subtypes were further reported in Table 1. The external cohort included 40 patients with histopathologically confirmed invasive breast carcinoma who underwent NAC and follow-up surgery after completing NAC. HER2 status was determined on biopsy specimens using HER2 IHC and/or FISH in accordance with the criteria of ASCO/CAP guidelines updated guidelines.

**Table 1:** Clinical and histopathological characteristics of HER2-positive and TNBC cases with neoadjuvant chemotherapy (NAC) in the study cohort and external validation cohort.

Cohort	Characteristics		Study cohort		External validation cohort	
			Case #/median	%/Range	Case #/median	%/Range
HER2+	Total case number		62	–	20	–
	Cases with residual tumor		24	38.71%	10	50.00%
	Cases with pCR		38	61.29%	10	50.00%
	Age (years)		56	30–76	43	30–69
	Nottingham grade	I	1	1.61%	0	0.00%
		II	27	43.55%	2	10.00%
		III	34	54.84%	18	90.00%
	Nuclear grade	I	0	0.00%	0	0.00%
		II	10	16.13%	1	5.00%
		III	52	83.87%	19	95.00%
	Estrogen receptor (ER) positive		30	48.39%	13	65.00%
	Progesterone receptor (PR) positive		19	30.65%	10	50.00%
	HER2/CEP17 ratio		6.73	1.23–22.98	7.00	0.96–11.10
	Residual cancer burden, if applicable		1.39	0.91–4.14	1.98	0.98–4.67
TNBC	Total case number		64	–	20	–
	Cases with residual tumor		37	57.81%	10	50.00%
	Cases with pCR		27	42.19%	10	50.00%
	Age (years)		51	26–74%	57	32–79%
	Nottingham grade	I	0	0%	1	5.00%
		II	15	23.4%	3	15.00%
		III	49	76.6%	16	80.00%
	Nuclear grade	I	0	0%	1	5.00%
		II	9	14.1%	2	10.00%
		III	55	85.9%	17	85.00%
	Residual cancer burden, if applicable		2.01	0.80–4.27	2.14	0.77–3.61

For complete study cohort metadata, please refer to Supplementary Information.

### Workflow and feature construction

**Fig. 1:**
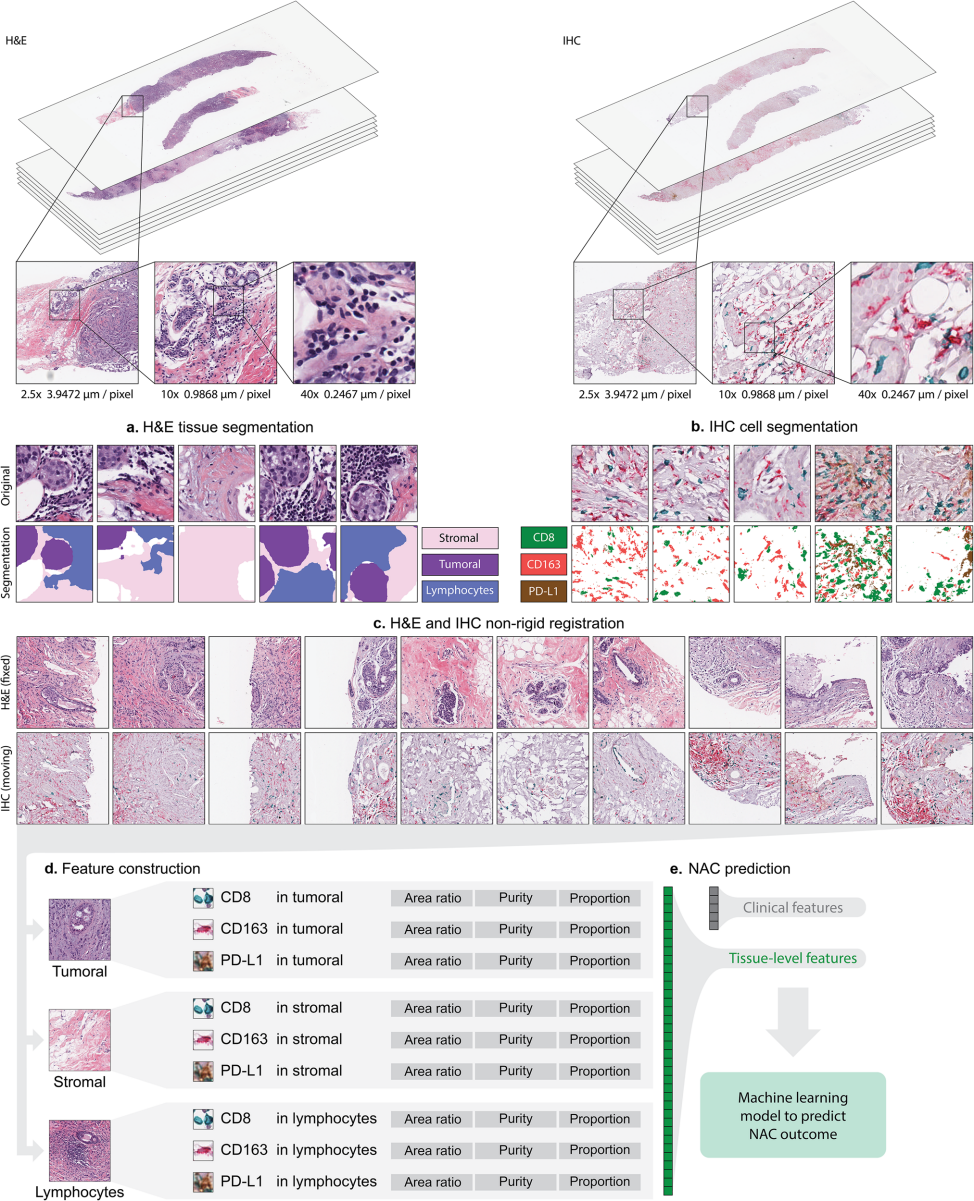
Overview of our workflow. **a** H&E tissue segmentation based on DeepLabV3 model. The segmentation generates stroma region, tumor region, and lymphocytes aggregated (lymph) region. **b** IHC markers segmentation. CD8, CD163, and PD-L1 were segmented. **c** H&E and IHC non-rigid registration. First row: representative H&E patches; second row: corresponding IHC patches after registration. **d** IMage-based Pathological REgistration and Segmentation Statistics (IMPRESS) feature construction. Totally 36 IMPRESS features were constructed. **e** Neoadjuvant chemotherapy (NAC) prediction with logistic regression.

**Fig. 2:**
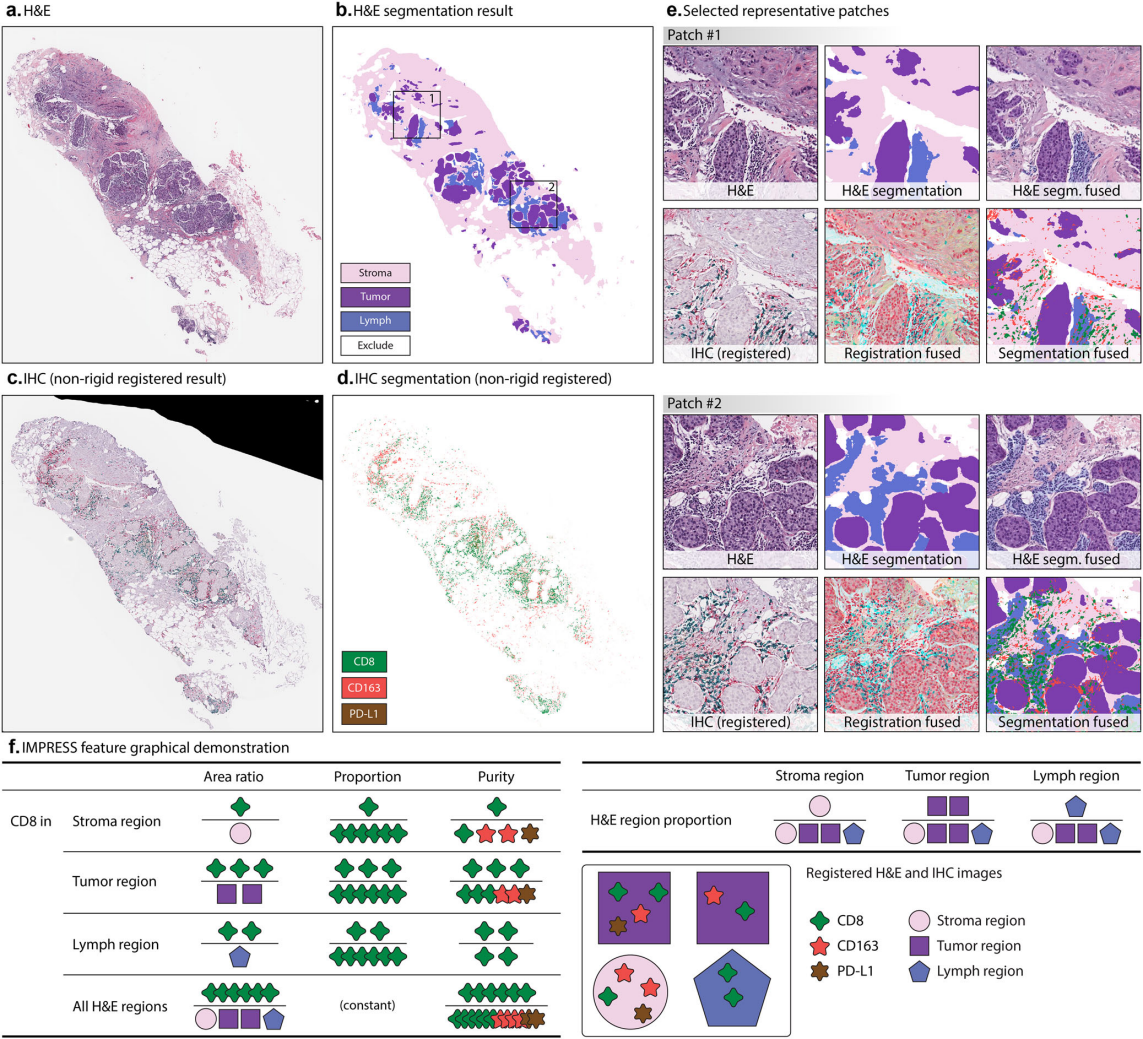
Tissue segmentation and image-level features extraction from registered H&E and IHC segmentation. **a** An example H&E tissue; **b** H&E tissue segmentation result; **c** IHC tissue (aligned to **a**) after non-rigid registration; **d** IHC segmentation results, after non-rigid registration. **e** Selected representative patches from **b** including (1) H&E patch, (2) H&E segmentation, (3) H&E segmentation (segm. in short) fused with original patch, (4) IHC patch after registration, (5) IHC patch after registration fused with H&E patch, and (6) H&E, IHC segmentation fused patch; **f** IMPRESS feature graphical demonstration. In **f**, each IHC marker produces 11 features (CD8 was shown as an example), H&E region produces 3 features, totally 36 IMPRESS features. Figure best viewed in color.

The workflow of this paper is presented in Fig. 1, including H&E image acquisition and segmentation, IHC image acquisition and segmentation, and H&E – IHC image registration. Given the input paired H&E and IHC WSIs, the automatic non-rigid registration was performed on each IHC WSI using the corresponding H&E WSI as fixed reference. With deep neural network “DeepLabV3” trained by pathologists labeled TCGA breast cancer H&E images^39^, H&E tissue segmentation was performed and four regions of interest were identified including stromal region (Stroma), tumoral region (Tumor), lymphocytes aggregated region (Lymph), and excluded region. All included regions (Stroma, Tumor, and Lymph) were defined as all H&E regions (All). Figure 2a, b shows an example of an H&E image and its segmentation result. The multiplexing IHC markers including CD8 (green), CD163 (red), and PD-L1 (brown) were identified via color-based K-means segmentation. Figure 2c, d shows an IHC image and its segmentation result. All results were reviewed and confirmed by two pathologists (A. Alkashash and C. Sancha).

Next, an AI-based automatic, accurate, comprehensive, interpretable, and reproducible WSI feature extraction pipeline was constructed, and generated 36 IMage-based Pathological REgistration and Segmentation Statistics (IMPRESS) features. Figure 2f demonstrates how IMPRESS features were calculated by using CD8 as an example. The full list of features is shown in Supplementary Table 1. The distribution of IMPRESS features’ expressions is demonstrated in Supplementary Figure 1 in violin plots with values ranging from 0 to 1.

In addition to IMPRESS features, clinical features and the status of molecular markers (ER, PR, and HER2) were exploited. In HER2+ cohort, six features were adopted including age, estrogen receptor status (ER+/−), estrogen receptor percentage (ER%), progesterone receptor status (PR+/−), progesterone receptor percentage (PR%), and the ratio of HER2 expression to chromosome 17 (HER2/CEP17). In TNBC cohort, age was the only available clinical feature since ER, PR, and HER2 were all negative.

### Machine learning model using IMPRESS features predicts NAC outcomes

LASSO-regularized logistic regression was adopted to evaluate the prediction power of the proposed IMPRESS features. In this study, four groups of features were compared, including all 36 IMPRESS plus clinical features (IMPRESS), IMPRESS H&E image features plus clinical features [IMPRESS (H&E only)] (Supplementary Table 1), IMPRESS IHC image features plus clinical features [IMPRESS (IHC only)] (Supplementary Table 1), and pathologists assessed IHC image features plus clinical features (Pathologists).

**Fig. 3:**
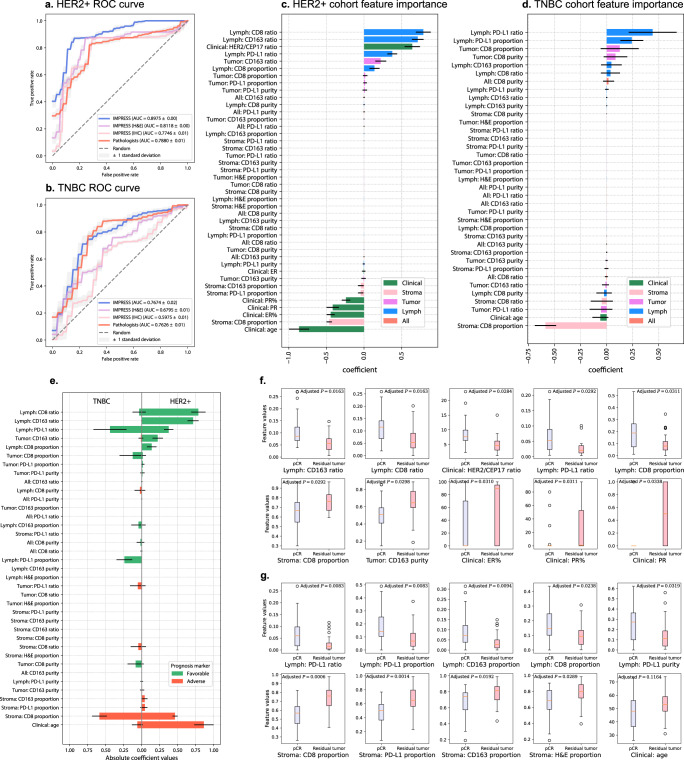
LASSO-regularized logistic regression machine learning model predicts NAC outcomes. **a**, **b** Receiver operating characteristic (ROC) curve for HER2+ (**a**) and TNBC (**b**) cohorts in the logistic regression results. Blue line: IMPRESS plus clinical features; Purple line: IMPRESS (H&E features only) plus clinical features; Pink line: IMPRESS (IHC features only) plus clinical features; Red line: pathologists assessed plus clinical features. **c**, **d** Feature importance generated by logistic regression. Positive coefficients are associated with better prognosis (pCR) and vice versa. Horizontal line in each bar stands for standard deviation. **c** HER2+ cohort; **d** TNBC cohort. **e** Comparison of IMPRESS and clinical coefficient importance in machine learning results between HER2+ and TNBC cohorts, organized by HER2+ coefficients in descending order. Coefficients in the horizontal bar plot were reported in absolute values, the positive values were defined as “favorable” prognostic markers and vise versa for negative values. Figure best viewed in colors. Horizontal line in each bar stands for standard deviation. **f**, **g** Univariate feature analysis in HER2+ cohort (**f**) and TNBC cohort (**g**) by comparing pCR cases against residual tumor cases. In **f** and **g**, top row showed five most favorable features, bottom row showed five most adverse features. Two-sided *P*-values were calculated based on Student’s *t*-test, followed with B&H procedure for multiple test adjustment (FDR = 0.05). For boxplot, the interior horizontal red line represents the median value, the upper and lower box edges represent 75^th^ and 25^th^ percentile, and the upper and lower bars represent the 90^th^ and 10^th^ percentiles, respectively.

We first compared IMPRESS with IMPRESS (H&E only) and IMPRESS (IHC only). From Table 2 and Fig. 3a, we found IMPRESS achieved significantly higher AUC than IMPRESS (H&E only; *t*-test statistic = 62.69, *P*-value = 5.68e-40) and IMPRESS (IHC only; *t*-test statistic = 79.97, *P*-value = 5.83e-44) in HER2 cases. Similarly, from Table 2 and Fig. 3b, we found IMPRESS achieved significantly higher AUC than IMPRESS (H&E only; *t*-test statistic = 16.87, *P*-value = 3.04e-19) and IMPRESS (IHC only; *t*-test statistic = 33.60, *P*-value = 7.23e-30) in TNBC cases. The results suggested that combining H&E and IHC histopathologic images can extract additional features for improved response to NAC prediction.

**Table 2:** LASSO-regularized logistic regression performances in HER2+ and TNBC cohorts.

Cohort	Features	AUC	F1 score	Precision (PPV)	Recall	NPV
HER2+	IMPRESS (all features)	**0.8975** ± **0.0038**	**0.8687** ± **0.0077**	0.8716 ± 0.0115	**0.8658** ± **0.0081**	**0.7897** ± **0.0110**
	IMPRESS (H&E only)	0.8118 ± 0.0048	0.8269 ± 0.0052	**0.9059** ± **0.0009**	0.7605 ± 0.0081	0.6977 ± 0.0070
	IMPRESS (IHC only)	0.7746 ± 0.0057	0.7775 ± 0.0085	0.8454 ± 0.0023	0.7197 ± 0.0129	0.6399 ± 0.0105
	Pathologists’ features	0.7880 ± 0.0065	0.7820 ± 0.0025	0.8696 ± 0.0061	0.7105 ± 0.0000	0.6446 ± 0.0026
TNBC	IMPRESS (all features)	**0.7674** ± **0.0209**	**0.7017** ± **0.0377**	**0.6903** ± **0.0286**	0.7148 ± 0.0552	0.7714 ± 0.0344
	IMPRESS (H&E only)	0.6795 ± 0.0103	0.5882 ± 0.0000	0.6250 ± 0.0000	0.5556 ± 0.0000	0.7000 ± 0.0000
	IMPRESS (IHC only)	0.5975 ± 0.0087	0.5915 ± 0.0103	0.5637 ± 0.0061	0.6222 ± 0.0152	0.7018 ± 0.0085
	Pathologists’ features	0.7626 ± 0.0095	0.6897 ± 0.0077	0.6454 ± 0.0135	**0.7407** ± **0.0000**	**0.7878** ± **0.0042**

*PPV* positive predictive value, *NPV* negative predictive value.

Experiments are repeated 20 times with different random seeds in leave-one-out cross-validation setting. mean value ± standard deviation are reported. Best performed mean values are highlighted in bold face.

### IMPRESS features outperformed pathologists’ assessed features for predicting NAC outcomes

Furthermore, IMPRESS features were compared to pathologists’ manually assessed IHCs image features for CD8, CD163, and PD-L1 and the details were described in the “Method” section.

In HER2+ cohort, we found IMPRESS achieved better performances (AUC = 0.8975 ± 0.0038) than Pathologists’ assessed features (AUC = 0.7880 ± 0.0065) significantly with *t*-test statistic = 64.59 (*P*-value = 1.84e-40; Fig. 3a). In TNBC cohort, we found IMPRESS achieved slightly better performances (AUC = 0.7674 ± 0.0209) than Pathologists’ assessed features (AUC = 0.7626 ± 0.0095) with *t*-test statistic = 0.94 (*P*-value = 3.54e-1; Fig. 3b). The detailed performances are summarized in Table 2. These results suggested that the AI-based features extracted from H&E and IHC histopathologic images can achieve equal or better performances than pathologists’ assessed features, and are the preferred input to develop machine learning algorithms to predict response to NAC in breast cancer patients.

The developed logistic regression models with all feature inputs were then directly applied to our external validation cohorts for HER2+ and TNBC subtypes, each with 10 pCR and 10 residual tumor patients. For HER2+ subtype, the AUC = 0.9005 ± 0.0060 (Supplementary Fig. 6a and Supplementary Table 8). For TNBC subtype, the AUC = 0.5865 ± 0.0157 (Supplementary Fig. 6b and Supplementary Table 8). Although a good AUC score was observed in HER2+ subtype, both models presented inadequate recall value (0.4000).

### Feature importance analysis in machine learning model

To systematically evaluate the pivotal features that dominate the prediction, we summarized the feature coefficients produced from LASSO-regularized logistic regression in Fig. 3c (HER2+ cohort) and Fig. 3d (TNBC cohort). The top important features are also summarized in Supplementary Table 5. For the HER2+ cohort, three out of the top five favorable prognostic markers (positively associated with pCR) were related to lymphocytes aggregated region, including CD8 ratio, CD163 ratio, and PD-L1 ratio. The favorable clinical prognostic marker of HER2/CEP17 ratio was ranked as the third, which echoes the finding in ref. ^40^ that suggested a high HER2/CEP17 ratio is significantly associated with pCR. In contrast, four out of the top five adverse prognostic markers (negatively associated with pCR) were related to clinical variables including age, ER ratio, PR positivity, and PR ratio. The second strongest adverse prognostic marker was *Stroma: CD8 proportion*. For the TNBC cohort, the top five favorable prognostic markers were *Lymph: PD-L1 ratio*, *Lymph: PD-L1 proportion*, *Tumor: CD8 proportion*, *Tumor: CD8 purity*, and *Lymph: CD163 proportion*. The top five adverse prognostic markers were *Stroma: CD8 proportion*, age, *Tumor: PD-L1 ratio*, *Stroma: CD8 ratio*, and *Lymph: CD8 purity*. Detailed feature importance ranking and coefficients are listed in Supplementary Table 5. From these results, we observed that features related to lymphocytes aggregated region (Lymph) were the most favorable prognostic markers to pCR. In addition, age, which plays an opposite role, is more critical in the HER2+ cohort than in the TNBC cohort. Interestingly, we found *Stroma: CD8 proportion* is one of the most adverse prognostic markers in both cohorts, suggesting more CD8 in the stromal region than in other regions is not a supportive sign for pCR.

The comparison of coefficient importance between HER2+ and TNBC cohorts is shown in Fig. 3e. Some IMPRESS features agreed in both HER2+ and TNBC cohorts. For example, *Lymph: PD-L1 ratio* and *Tumor: CD8 proportion* act as common favorable features to pCR; Age and *Stroma: CD8 proportion* act as common adverse features to pCR. However, we also observed some disparities between the HER2+ and TNBC cohorts: CD8 and CD163 played more essential roles in HER2+ cohort (*e.g*., *Lymph: CD8 ratio* and *Lymph: CD163 ratio*), whereas PD-L1 was more informative in the TNBC cohort. Similar results can also be observed in the following univariate analysis (Fig. 3f, g).

### Univariate analyses with NAC response

As AI-based IMPRESS features outperformed pathologists’ assessed features in predicting pCR and were correlated with RCB, we further performed univariate analyses to investigate the relationships between IMPRESS features and NAC responses and to identify specific IMPRESS features which showed significant differences in predicting NAC response between the HER2+ and TNBC cohorts.

We compared each feature by using pCR cases against residual tumor cases using the two-sided Student’s *t*-test. The top five favorable/adverse features with the most significant differences are presented in Fig. 3f for the HER2+ cohort and in Fig. 3g for the TNBC cohort. Complete results are further presented in Supplementary Table 6. We found that the most significantly different features in pCR cases against residual tumor cases are highly consistent with those identified by machine learning methods, such as *Lymph: CD163 ratio* (adjusted *P*-value = 0.0163) and *Lymph: CD8 ratio* (adjusted *P*-value = 0.0163), two top-ranked favorable features for HER2+ cases, which were identified by both univariate analysis and machine learning model. Nevertheless, a few features identified by the univariate analysis were not concord with machine learning results. For example, *Tumor: CD163 purity* (adjusted *P*-value = 0.0298), one of the adverse features in HER2+ cases, was not identified in machine learning (Fig. 3c). Similar inconsistencies were also found in TNBC cases, such as *Lymph: CD8 proportion* (adjusted *P*-value = 0.0238).

To present an alternative point of view of the relationship between IMPRESS features and pCR, Spearman’s rank correlation coefficient (SCC) was used to evaluate the differences among the features regarding their relationship to pCR. The results were shown in Supplementary Fig. 4a (HER2+) and Supplementary Fig. 4b (TNBC). The SCC results were largely consistent with the machine learning feature importance results (in Fig. 3c, d) and the univariate analysis results (in Fig. 3f, g), especially for the features related to lymphocytes aggregated regions and tumoral regions. These results confirmed the important roles of pre-NAC TILs in predicting pCR.

Furthermore, we found that several IMPRESS features expressed significantly higher in HER2+ than in TNBC based on the Mann–Whitney *U* test results in Supplementary Figure 3, such as *Stroma: PD-L1 purity* (adjusted *P*-value = 1.02e-3), *Lymph: CD163 ratio* (adjusted *P*-value = 1.02e-3), *etc*. Some features expressed significantly lower in HER2+ than TNBC, such as *Tumor: CD8 purity* (adjusted *P*-value = 2.31e-3), *Stroma: CD8 purity* (adjusted *P*-value = 2.31e-3), *etc*. These results suggested that IMPRESS features distributed differently among different breast cancer cohorts, providing a different perspective between two breast cancer subtypes.

### Relationships between IMPRESS features and residual cancer burden

**Fig. 4:**
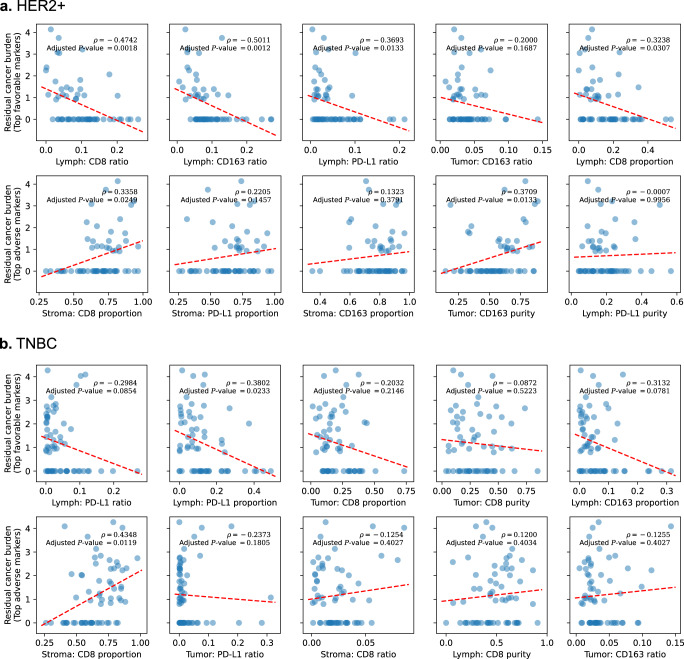
Scatter plot with Spearman’s rank correlation coefficient *ρ* and *P*-value between IMPRESS features and residual cancer burden (RCB). **a** HER2+ cohort, first row: top 5 favorable IMPRESS features; second row: top 5 adverse IMPRESS features; **b** TNBC cohort, first row: top 5 favorable IMPRESS features; second row: top 5 adverse IMPRESS features. Dashed red lines represent the fitted linear regression slopes. All *P*-values were adjusted with B&H procedure (FDR = 0.05).

In addition to pCR, residual cancer burden (RCB) was calculated in patients with residual tumor. The median RCB in the HER2+ cohort is 1.39 with a range of 0.91–4.14. The median RCB in the TNBC cohort is 2.01 with a range of 0.80–4.27. RCB was defined as 0 for patients with pCR. The non-parametric statistics from SCC *ρ* with two-sided *P*-values were used to examine the relationships between IMPRESS features and RCBs. The top 5 most favorable and most adverse IMPRESS prognostic features from machine learning analyses listed in Supplementary Table 5 were further compared with RCBs [Fig. 4a (HER2+) and Fig. 4b (TNBC)]. The complete list is showed in Supplementary Table 7.

As demonstrated in Fig. 4a, HER2+ cases showed *Lymph: CD8 ratio*, *Lymph: CD163 ratio*, *Lymph: PD-L1 ratio*, and *Lymph: CD8 proportion* were negatively correlated with RCB significantly. In contrast, *Stroma: CD8 proportion* and *Tumor: CD163 purity* were positively correlated with RCB significantly. From the TNBC cohort in Fig. 4b, *Lymph PD-L1 proportion* was negatively correlated with RCB significantly. In contrast, *Stroma: CD8 proportion* was positively correlated with RCB.

One study by Meisel et al.^40^ suggested that TILs associated with RCB in HER2+ subtype breast cancer NAC patients. In our results, we further demonstrated the association between TILs and RCB using correlation analysis, especially the *Lymph: PD-L1 ratio*. Furthermore, the inverse relations were detected between RCB scores and CD8 + TIL in Miyashita et al.^41^, which also agreed with our findings such as *Lymph: CD8 ratio* and *Lymph: CD8 proportion* in the HER2+ subtype (Supplementary Table 7).

These results suggested that the AI-based IMPRESS features from pre-NAC images can also predict RCB values in a quantitative manner. For example, *Lymph: PD-L1 ratio* (favorable marker) and *Stroma: CD8 proportion* (adverse marker) were two common features that were significantly or highly correlated with RCB in both the HER2+ and TNBC cohorts.

### Correlation analyses disclose latent dependencies in IMPRESS features

**Fig. 5:**
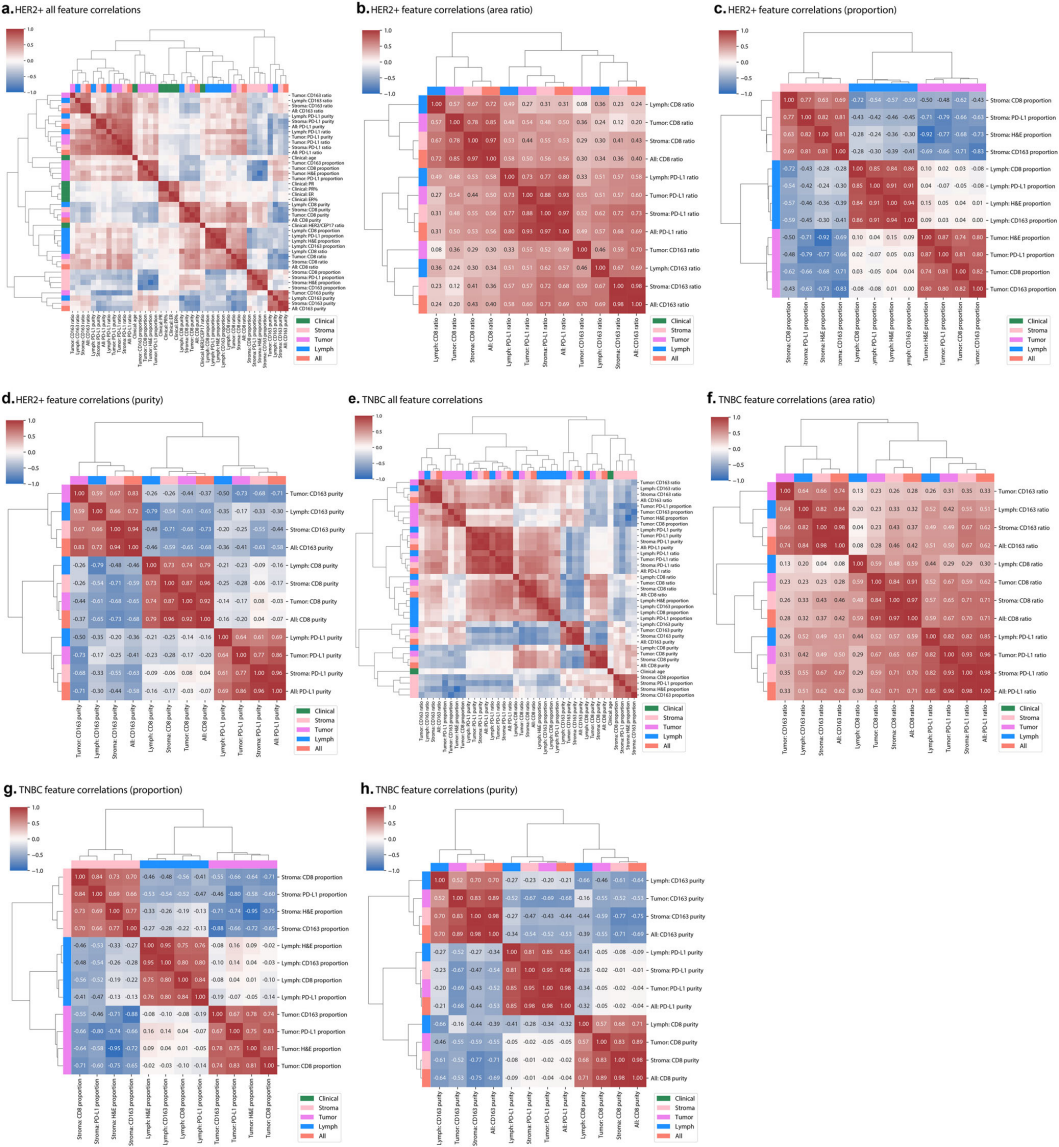
Correlation analyses for IMPRESS features in HER2+ and TNBC cohorts. **a** HER2+ all IMPRESS feature correlation matrix; **b** HER2+ area ratio correlation matrix; **c** HER2+ proportion correlation matrix; **d** HER2+ purity correlation matrix; **e** TNBC all IMPRESS feature correlation matrix; **f** TNBC area ratio correlation matrix; **g** TNBC proportion correlation matrix; **h** TNBC purity correlation matrix. Figure best viewed in color.

To fully investigate the relationships and unveil the latent dependencies among IMPRESS features, pair-wised SCC were analyzed (Fig. 5). These pair-wised SCC *ρ* demonstrated the latent relationships between each pair of IMPRESS features. The overall feature correlations present subtle differences between HER2+ cohort (Fig. 5a) and TNBC cohort (Fig. 5e).

All SCC *ρ* for area ratio features were positive. We were particularly interested in those highly correlated area ratio features from different IHC markers. For area ratio in HER2+ (Fig. 5b), the most correlated ratio statistics from different IHC markers were *Stroma: PD-L1 ratio* and *All: CD163 ratio* (*ρ* = 0.73, *P*-value = 2.29e-11); *Stroma: CD163 ratio* and *Stroma: PD-L1 ratio* (*ρ* = 0.72, *P*-value = 6.64e-11). For area ratio in TNBC (Fig. 5f), the most correlated ratio statistics from different IHC markers were *Stroma: PD-L1 ratio* and *Stroma: CD8 ratio* (*ρ* = 0.71, *P*-value = 3.82e-11); *All: PD-L1 ratio* and *Stroma: CD8 ratio* (*ρ* = 0.71, *P*-value = 3.64e-11); *All: PD-L1 ratio* and *All: CD8 ratio* (*ρ* = 0.71, *P*-value = 6.88e-11). The results of area ratio statistics suggested that the area ratio of PD-L1 had the strongest association with CD163 in HER2+, but had the strongest association with CD8 in TNBC.

For the proportion statistics in IMPRESS features, positive correlations were observed within same H&E regions. In contrast, negative correlations were observed across different H&E regions (Fig. 5c, d). We were particularly interested in those features that from different H&E regions with most negative correlations. In HER2+ (Fig. 5c), the most negatively correlated proportion statistics were *Tumor: H&E proportion* and *Stroma: H&E proportion* (*ρ* = −0.92, *P*-value = 1.57e-25); *Tumor: CD163 proportion* and *Stroma: CD163 proportion* (*ρ* = −0.83, *P*-value = 4.18e-17). In TNBC (Fig. 5g), the most negatively correlated proportion statistics were *Tumor: H&E proportion* and *Stroma: H&E proportion* (*ρ* = −0.95, *P*-value = 3.01e-33); and *Tumor: CD163 proportion* and *Stroma: CD163 proportion* (*ρ* = −0.88, *P*-value = 2.52e-21). The results of proportion statistics suggested that CD163 was the most negatively correlated IHC marker populated at either tumoral or stromal region.

For the purity statistics in IMPRESS features, positive correlations were observed within same IHC markers. In contrast, negative correlations were observed across different IHC markers (Fig. 5d, h). We were particularly interested in those features that from different IHC markers with most negative correlations. In HER2+ (Fig. 5d), the most negatively correlated purity statistics from different IHC markers were *Lymph: CD163 purity* and *Lymph: CD8 purity* (*ρ* = −0.79, *P*-value = 1.96e-14); *Stroma: CD163 purity* and *All: CD8 purity* (*ρ* = −0.73, *P*-value = 1.05e-11); *Tumor: CD163 purity* and *Tumor: PD-L1 purity* (*ρ* = −0.73, *P*-value = 2.46e-11). In TNBC (Fig. 5h), the most negatively correlated purity statistics from different IHC markers were *Stroma: CD163 purity* and *Stroma: CD8 purity* (*ρ* = −0.77, *P*-value = 7.85e-14); *Stroma: CD163 purity* and *All: CD8 purity* (*ρ* = −0.75, *P*-value = 7.18e-13). The results of purity statistics suggested that CD163 and CD8 were two most distinct IHC markers that populated against each other among various H&E regions.

## Discussion

Recently, AI-based computational pathology methods based on tumor morphology have been developed to predict the clinical outcome including survival^5,6^. Additionally, evaluating cell-level features in tumor immune micro-environment such as tumor-infiltrating lymphocyte (TIL) in pre-treatment breast cancer biopsies to predict NAC outcomes is also imperative and can contribute to potential clinical guidelines and treatment intervention. To the best of our knowledge, this is the unique study to provide a whole slide image (WSI) feature extraction pipeline to quantitatively evaluate the histopathological features extracted from both H&E-stained and IHC-stained WSIs and predict NAC outcomes using machine learning model based on features derived from tumor itself and tumor immune micro-environment.

This study has several strengths and advantages. First, analyzing histopathologic images is one of the most challenging machine learning tasks, hindered by the large size of the microscopy images^42^. Studies usually sliced WSIs into several small patches^42^, but different choices of patch sizes can increase the uncertainties of models and performances. In this paper, the AI-based IMPRESS features were assessed on the WSI level, which is a more robust and reproducible feature extraction pipeline. Second, this paper fully utilized the paired H&E-stained and IHC-stained WSIs. Since the image registration process was proved to be robust and accurate, the extra information derived from IHC-stained WSIs can provide detailed tumor immune micro-environment information complementary to the tumor H&E images. Different from other methods that solely relied on H&E WSIs to extract lymphocytes, the identifications of CD8, CD163, and PD-L1 provide extra information, which help us better characterize the tumor immune micro-environment. Third, AI is suggested to be an automatic approach for providing a potential clinical guideline. However, many AI-based methods are limited by their poor interpretability and unpredictable performance, especially when end-to-end learning methods were used. Our experiments not only demonstrated that the AI-based automatic feature extraction pipeline has the capacity to generate interpretable IMPRESS features, but can also predict NAC outcome equally or more accurate than the model based on pathologists’ assessed features. Last but not the least, many feature extraction methods were based on pathologists’ manual assessments (e.g., Ali et al.^15^ Hwang et al.^16^). The features assessed by pathologists conveyed rich interpretable explanations, however, they were difficult to reproduce with consistent quality. Instead, our automatic feature extraction pipeline produced abundant reproducible interpretable features (36 IMPRESS features), and also proved to outperform pathologists’ assessment in HER2+ cohort (or have equal performances in TNBC cohort) using the logistic regression model.

In current study, we also investigated the association of clinicopathologic features from pre-treatment biopsies with response to NAC in two different breast cancer subtypes, HER2+ BC and TNBC. Previous study^43^ found that the increased TIL concentration can predict response to neoadjuvant chemotherapy and survival but differences were observed between HER2+ and TNBC subtypes. In our results, we found several common and different feature behaviors across those two breast cancer subtypes, suggesting that breast cancer is immunogenic^43^ and TILs might target differently in different breast cancer subtypes.

Our study has also demonstrated the relationship between several tumor immune micro-environment features and pCR. One of the most interesting findings is PD-L1 expression in pre-treatment tumor immune micro-environment, especially in TNBC cohort. It has been reported that the upregulation of PD-L1 is involved in various cellular processes in cancer cells as well as interactions between cancer cells and immune cells^44–46^. It has been conflicting whether PD-L1 expression is a favorable or adverse prognostic factor for breast cancer patients’ survival^47–54^. The conflicting conclusions may result from the differences in composition of cohorts, PD-L1 antibody clones, or assessment methods (most studies used manual assessment). In our study, PD-L1 in lymphocytes aggregated region was found to associate with a favorable response to NAC. Kong et al.^55^ suggested that PD-L1 expression at different locations had different impacts on survival in colorectal cancer (CRC) patients, and showed that total PD-L1 expression was a favorable prognostic marker. In our study, we observed similar behavior of high TIL and PD-L1 expression.

Furthermore, our data has also demonstrated that the most important IMPRESS features identified from the logistic regression model to predict pCR (such as CD8, CD163, and PD-L1 ratios in lymphocytes aggregated region, and CD8 proportion in lymphocytes aggregated region) also correlated with RCB, at least partially. The correlation analyses for IMPRESS features to themselves (Fig. 5) revealed the highly and densely correlated features, providing additional insights to morphologic and clinical features which are important for therapy response in breast cancers. The correlation analyses for IMPRESS features to residual cancer burden (RCB; Supplementary Table 7) found more significant features in HER2+ subtype (13 out of 36) than in TNBC subtype (3 out of 36), suggesting IMPRESS features may well characterize those residual tumors in HER2+ breast cancer patients.

Several limitations remained in our study. First, due to the limitation of the data source, the size of the cohorts is relatively small. Second, the markers from IHC-stained WSIs were limited to CD8, CD163, and PD-L1, which may not represent the entire tumor immune micro-environment. In light of our results demonstrating that AI-based IMPRESS features derived from pre-treatment H&E and IHC histopathologic images can predict pCR outcome, we would expect to see advanced machine learning studies with additional immune IHC markers in the future, such as Ki67 or other proliferation-associated markers. Third, TNBC cohort is not performing that well in study cohort (0.7674 ± 0.0209), and had deteriorated sharply in its external validation cohort (0.5865 ± 0.0157). Encouraged by the facts that one-fourth of the IMPRESS features were significantly differentially valued between pCR and residual tumor cases (Supplementary Table 6), we argue that the poor performance on TNBC cohort may be partially due to the limited size of the study samples, and thus a simple linear machine learning model was not capable to learn sample heterogeneity. Compared with consistently good performances on both the study cohort and the external validation cohort in HER2+ subtype, it is also reasonable to suspect that the TNBC cohort may harbor more heterogeneous and complex tumor micro-environments than the HER2+ subtype. In addition, we observed fair performances of negative predictive value (NPV) for HER2+ (study cohort: 0.7897 ± 0.0110, external validation: 0.6250 ± 0.0000) and suboptimal NPV for TNBC (study cohort: 0.7714 ± 0.0344, external validation: 0.5582 ± 0.0166). These NPV values suggest a considerable amount of false negatives, which is insufficient to let the current model be in use in a clinical setting. If more cases will be available, it is possible to train a more robust and sophisticated machine learning model for the task and finally become a useful clinical triage tool. Fourth, combining H&E and IHC stain images requires an extra step of registration. Since they are not from the same glass slide, nuclei are thus not one-to-one mapped. Given the current technologies of multiplex slide images such as Multiplex Ion Beam Imaging (MIBI) or co-detection by indexing (CODEX), or even spatial transcriptomics, the prediction of post-NAC outcomes can potentially be improved. Lastly, although IMPRESS features can well characterize pCR and residual tumor cases, the features are on the basis of whole slide image statistics. If one can extract regional IMPRESS features from certain types of micro-environments, it would be more clinically useful as pathologists can trace back to a certain region that leads to a bad outcome.

Compared to the classic clinical scores, AI can objectively evaluate slides, not to mention the recent commercialization in digital pathology. As further depicted by a pathology startup Okwin^56^ in 2022, AI helps discriminate high and low risk of relapse for early ER + HER2- breast cancer. These emerging AI tools can enable an early rule-out with a decent amount of the cases. However, as we shown from the TNBC cohort, the power of AI may limited to several, not all, cancer subtypes. Thus, the prognosis power we presented on HER2+ and TNBC subtypes may further benefit the future research endeavors, including but not limited to power analysis and model selection.

In summary, we constructed an automatic, accurate, comprehensive, interpretable, and reproducible WSI feature extraction pipeline (IMPRESS) and used these IMPRESS features to develop machine learning model to predict the response to NAC in breast cancer patients. The machine learning models used combined feature sets showed promising performances, especially for HER2+ subtype. The univariate analyses identified pCR-associated and RCB-associated image features, these tumor immune micro-environment signals can be either served as predictive markers or used for refining the choice of first-line treatment, which can potentially be essential players toward precision oncology.

## Methods

### Hardware and software

This study was approved by the Ohio State University Institutional Research Board. Written informed consent was obtained from all individual patients included in the study.

All experiments were conducted on a high-performance computing cluster. In particular, we took advantage of four NVIDIA V100 graphics processing units (GPUs) and 1.6TB local storage. We used OpenSlide^57^ (version 1.1.2) to access the WSI files, and PyTorch (version 1.6.0, torchvision version 0.7.0) for data loading, model training and testing. Machine learning and statistical analyses were performed in python with scikit-learn (version 0.23.2). We used pillow (version 7.2.0) and OpenCV (version 4.4.0) for image processing in python. We used pandas (version 1.0.5) for data processing.

### Patients and specimens

This study included 62 HER2-positive breast cancer (HER2+) female patients and 64 triple-negative breast cancer (TNBC) female patients treated with neoadjuvant chemotherapy (NAC) and follow-up surgical excision. In accord with STARD-2015 guideline, patients with histopathologically confirmed invasive breast carcinoma who underwent NAC from January 2011 to December 2016, those who had underwent surgery after completing NAC were included. HER2 status was determined on biopsy specimens using HER2 IHC and/or fluorescence in situ hybridization (FISH) in accordance with the criteria of American Society of Clinical Oncology (ASCO)/College of American Pathologist (CAP) guidelines updated guidelines^58^. In addition, two sets of external validation datasets were further used to evaluate the developed machine learning model (20 for HER2+, 20 for TNBC, each with 10 pCR cases, 10 residual tumor cases).

### Pathologic assessment of the response to neoadjuvant chemotherapy

For neoadjuvant chemotherapy, all HER2+ patients received four cycles of AC (doxorubicin/cyclophosphamide) together with Taxol (paclitaxel/docetaxel) and trastuzumab except 7 patients (3 with residual tumor, 4 without residual tumor) who received four cycles of AC together with PTD (pertuzumab + trastuzumab + docetaxel). Triple-negative breast cancer patients received AC (doxorubicin/cyclophosphamide) together with Taxol (paclitaxel/docetaxel).

After NAC, all study cohort patients underwent surgery and the resection specimens were examined grossly and microscopically. A pathologic complete response (pCR) was defined as no detectable residual invasive carcinoma (excluding in situ carcinoma) and absence of any metastatic tumor in lymph node, while the presence of residual invasive carcinoma in breast or in lymph node designated the incomplete response.

Residual cancer burden (RCB) was assessed in all cases with incomplete response by comparing the pre-treatment core needle biopsy with the post-treatment resection specimen. RCB value was calculated based on tumor cellularity, tumor size change, and lymph node metastasis as described previously^59^.

### Multi-color multiplex immunohistochemistry with CD8, CD163, PD-L1, and assessment by pathologists

Multi-color multiplex immunohistochemistry (IHC) with CD8 for cytotoxic T lymphocytes (clone MRQ26, mouse, Ventana), CD163 for macrophages (clone SP57, rabbit, Ventana), and PD-L1 (clone SP263, rabbit, Ventana) was performed on freshly cut whole sections from pretreatment biopsies as described before^60,61^. A membranous PD-L1 staining in tumor cells or immune cells was considered as specific staining. The immunohistochemistry was evaluated with consensus viewing by two pathologists (Y. Hou and Z. Li). The percentage of PD-L1 positively-stained cells were recorded and used for machine learning models (features that generated by pathologists). The parameters assessed were as follows: PD-L1 expression in tumor cells (PD-L1 TC), PD-L1 expression in immune cells (PD-L1 IC), PD1 expression in immune cells, intratumoral CD8+ immune cells (IT-CD8+), peritumoral CD8+ immune cells (PT-CD8+), intratumoral CD163+ macrophages (IT-CD163+), and peritumoral CD163+ macrophages (PT-CD163+).

### Non-rigid image registration

All H&E-stained and IHC-stained slides were scanned into WSIs using Hamamatsu scanner with 20x magnification. Although H&E-stained slides and IHC-stained slides from each case are continuous sections from paraffin-embedded tissue blocks, they were not always well aligned in the same space (2-D Euclidean space). In order to correctly assemble CD8 cytotoxic T-cells, CD163 macrophages, and PD-L1-expressing cells on H&E stained images, non-rigid image registration was applied on IHC-stained images using H&E-stained images as templates.

Specifically, we adopted a multi-step, automatic, and non-rigid histological image registration method^62,63^ and applied it to our dataset. First, the images were converted into grayscale, downsampled, and histogram equalized. Then an initial rigid registration was performed. Next, a non-rigid registration was performed. The algorithm automatically selected the best nonrigid transformations according to various versions of demons algorithms^64^, local affine registration^65^, or a feature-point-based thin-plate spline interpolation. A few tissues in WSIs which had visually bad registration results were excluded.

### H&E region segmentation

The H&E region segmentation aims to automatically identify the stromal tissue region, tumoral tissue region, and lymphocytes aggregated tissue region. In this paper, we fully utilized the breast cancer dataset from The Cancer Genome Atlas (TCGA)^66^ consisting of 151 images^39^ as training data, where each image has a segmentation map with 22 region classes labeled by multiple pathologists. We sliced those images with 10% horizontal and vertical overlapping, and generated 900 patches in total. Each patch is in 20× magnification (around 0.5 micron per pixel) with 1024×1024 pixels in size.

We defined four segmentation classes, including (1) stromal region (Stroma), (2) tumoral region (Tumor), (3) lymphocytes aggregated region (Lymph), and (4) excluded region (Exclude). The tumoral region includes invasive carcinoma and angioinvasion regions. The lymphocytes aggregated region includes lymphocytic infiltration, lymphatics, and other immune infiltrate, as well as considering the inflammation-rich area. The excluded region contains background or other regions not of our interest (*e.g.*, adipocytes).

The deep learning model “DeepLabV3” (https://arxiv.org/abs/1706.05587) was adopted to learn the segmentation of the H&E regions. In DeepLabV3, “atrous convolution” was introduced and has the ability to capture larger field-of-view as well as control the resolution of feature responses. In detail, the residual network ResNet-101 (https://arxiv.org/abs/1512.03385) was employed into DeepLabV3 and was implemented in PyTorch and torchvision with auxiliary loss weight = 0.5. During the training, weighted mean squared error loss criterion was used by adopting the inverse of number of the pixels as class weights. Adaptive moment estimation (Adam) optimizer (https://arxiv.org/abs/1412.6980) was adopted with learning rate = 1e-4 and batch size = 2 throughout the experiments. We searched the number of epochs as the hyper-parameter^67^ with five-fold cross-validation training scheme. Dice coefficients

$$Dice = \frac{{2 \times TP}}{{(TP + FP) + (TP + FN)}}$$

was adopted to evaluate the model performance, where TP stands for true positive pixels, TN stands for true negative pixels, FP stands for false positive pixels, and FN stands for false negative pixels, respectively. A higher dice coefficient suggests a better performance.

We split 900 image patches into training, validation, and testing sets. We firstly held out 10% of the image patches for testing (these patches were not used for any training purposes). Next, five-fold cross-validation training scheme was applied to the rest of the 810 patches. Basically, in each fold, 80% of the data were used for training, and 20% of the data were used for validation (i.e., tuning the hyper-parameter). Patches cropped from the same image will not be separated into different sets. Models were evaluated every 20 epochs, the optimal number of epochs was chosen according to the optimal mean dice coefficients among the five folds. We found the number of epochs = 280 gives the optimal validation performances. After the optimal number of epochs was determined, the deep learning model were applied on the entire training set for model training, and report the testing performances on the 10% held out testing set.

All performances were measured in dice coefficients. The final training performances are 0.9881 for stromal region, 0.9941 for tumoral region, 0.9876 for lymph region, and 0.9911 for excluded region. The mean dice coefficient for training is 0.9902. The final testing performances are 0.8314 for stromal region, 0.8880 for tumoral region, 0.7065 for lymph region, and 0.7996 for excluded region. The mean dice coefficient for testing is 0.8064.

Finally, the trained DeepLabV3 model was then applied to our study cohorts HER2+ and TNBC. The trained TCGA images and the targeted HER2+ and TNBC WSIs are in same magnifications (20× objective lens). We firstly sliced H&E WSIs into 1024×1024 pixels patches with 200 pixels horizontal and vertical overlapping. Then, during the feed-forward process in deep neural networks, the predicted class probabilities in each pixel at overlapped regions were averaged, and the class with highest probability in each pixel was voted as the prediction result.

### Immunohistochemistry markers segmentation

Segmenting the IHC markers including CD8, CD163, and PD-L1, which amplified by several visually distinctive colors, is one of the essential step for acquiring final image features. In this study, Color-based K-means clustering was performed to segment CD8, CD163, PD-L1, and other areas (background and area not of interest).

Firstly, at most 10 image patches with 512×512 pixels in size were selected from each IHC tissue with lowest excluded region ratio (from H&E segmentation results) in HER2+ and TNBC cohorts, respectively. Secondly, we convert all selected image patches from RGB color space to L*a*b* color space, which ensures the highest color contrast across three different IHC markers. Thirdly, a K-means clustering was performed and aggregates each pixels of selected patches in L*a*b* color space. In detail, we set *K* = 15, number of initialization = 3, and maximum number of iteration = 300 with tolerance = 1e-4. For external validation, we remained the same parameter setting except *K* = 30.

We compared each four 1024×1024 patches from HER2+ and TNBC cohorts with two pathologists manually labeled IHC markers, the dice coefficients were then reported.

### IMPRESS feature extraction

In total 36 image-based features were extracted from the proposed IMPRESS pipeline (Fig. 2f). All features were calculated based on the WSI from each patient. Basically, each of CD8, CD163, and PD-L1 IHC markers will produce 11 features, which are the combination of “area ratio” (or “ratio” in short), “proportion”, “purity” in *Stroma*, *Tumor*, *Lymph*, and *All* H&E regions. Here *Lymph* stands for lymphocytes aggregated region. The proportion in *All H&E* regions were excluded as it always equals to 1. In addition, 3 features from H&E region proportions were also exploited: (1) the ratio of stromal region to all H&E regions; (2) the ratio of tumoral region to all H&E regions; and (3) the ratio of lymphocytes region to all H&E regions. Thus the total number of IMPRESS features is 3 × 11 + 3 = 36.

The definition of the area ratio (*e.g*., *Lymph: CD8 ratio*) is the ratio of the total number of pixels of an IHC marker (CD8) on a certain H&E region (lymph) to the total number of pixels of that H&E region (lymph). The area ratio can be interpreted as how much of an IHC marker can be expressed on a certain type of tumor microenvironments. The definition of the proportion (*e.g*., *Lymph: CD8 proportion*) is the ratio of the total number of pixels of an IHC marker (CD8) on a certain H&E region (lymph) to the total number of pixels of that marker (CD8) on all valid H&E regions. The definition of the purity (*e.g*., *Lymph: CD8 purity*) is the ratio of the total number of pixels of an IHC marker (CD8) to the total number of pixels of all IHC markers (CD8, CD163, and PD-L1) on a certain H&E region (*e.g.*, *Lymph*).

The definition of “all H&E regions” (*All*) is the pixel sum of stroma, tumor, and lymphocytes aggregated regions. The full list of features was also presented in Supplementary Table 1.

### Reliability and results of IMPRESS feature extraction pipeline

The H&E tissue segmentation produced four regions of interests: stromal region (Stroma), tumoral region (Tumor), lymphocytes aggregated region (Lymph), and exclude region (Exclude). Each cohort has pathologist labeled 25 patches in 20× magnification, each with 512×512 pixels. The dice coefficient in HER2+ cohort for each class is 0.9312 (stromal region), 0.8413 (tumoral region), 0.7035 (lymphocytes aggregated region), and 0.8482 (exclude region). The mean dice coefficient in HER2+ cohort is 0.8311. The dice coefficient in TNBC cohort for each class is 0.9140 (stromal region), 0.7576 (tumoral region), 0.7323 (lymphocytes aggregated region), and 0.8752 (exclude region). The mean dice coefficient in TNBC cohort is 0.8198. The confusion matrices for HER2+ and TNBC cohorts were also reported in Supplementary Table 2.

The IHC marker segmentation produced four regions of interest: CD8 region, CD163 region, PD-L1 region, and exclude region. Each cohort has pathologist labeled 5 patches in 20× magnification, each with 512×512 pixels. The dice coefficient in HER2+ cohort for each class is 0.8422 (CD8 region), 0.7379 (CD163 region), 0.7669 (PD-L1 region), and 0.9506 (exclude region). The mean dice coefficient in HER2+ cohort is 0.7823. The dice coefficient in TNBC cohort for each class is 0.8608 (CD8 region), 0.7500 (CD163 region), 0.7237 (PD-L1 region), and 0.9693 (exclude region). The mean dice coefficient in TNBC cohort is 0.7782. The confusion matrices for HER2+ and TNBC cohorts were also reported in Supplementary Table 3.

A non-rigid registration process was performed on each tissue for every pair of tissues in WSI. The total number of pathologists labeled landmark correspondences were 516 for HER2+ cohort and 304 for TNBC cohort. The evaluation performances including mean and median of the distance (in *µm*) and median *rTRE*^62^ before and after registration were reported in Supplementary Table 4. We consider the registration is adequate if the median distance (*µm*) is ≤50 *µm*. From the results, we found the distances before and after registration in HER2+ cohort are 374.01 *µm* and 33.31 *µm* in mean, or 278.73 *µm* and 18.23 *µm* in median. The distances before and after registration in TNBC cohort are 627.66 *µm* and 47.78 *µm* in mean, or 48.14 *µm* and 27.13 *µm* in median. Both results in HER2+ and TNBC cohorts suggest the paired pathology images were aligned adequately. An example H&E tissue and the corresponding IHC tissue with 36 landmark correspondence pairs were demonstrated in Supplementary Fig. 2A, B.

The top IMPRESS features with favorable or adverse prognostic values were further exploited with their associated image patches. In each cohort, the cases with highest image feature value were selected, and their WSIs were sliced into patches with 1024×1024 pixels. Representative patches were presented within that specific patient’s WSI and were shown in Supplementary Fig. 5. Supplementary Fig. 5A presented representative patches in HER2+ with top important features; Supplementary Fig. 5B presented representative patches in TNBC with top importance features. The adverse prognostic markers were highlighted in gray backgrounds. These results helped to visualize typical image patches where the top important features were enriched.

### Machine learning settings for NAC outcome prediction

Due to the sample size, leave-one-out training and testing scheme was adopted. Given N patients in the data cohort, each time 1 patients were held out for testing, and the remained N-1 patients were used for training and validation. For the N-1 patients during training, five-fold cross-validation was adopted. For each fold, 80% of the data was used for training, and the rest 20% data was used for model validation (*i.e*., finding the hyper-parameters of the model). All features in training & validation set were standardized to standard normal distribution, and the standardization was also applied to the testing set.

Logistic regression model implemented in scikit-learn (version 0.23.2) was adopted to predict NAC outcome. The objective function for LASSO-regularized logistic regression is

$$Minimize\;L(\theta ) = \frac{1}{{N - 1}}\left[ {\mathop {\sum}\nolimits_{i = 1}^{N - 1} { - \left( {\alpha _1y^{(i)}log\left( {h_\theta \left( {x^{(i)}} \right)} \right) + \alpha _2\left( {1 - y^{(i)}} \right)log\left( {1 - h_\theta \left( {x^{(i)}} \right)} \right)} \right)} + \lambda \mathop {\sum}\nolimits_{j = 1}^K {\left| {\theta _j} \right|} } \right],$$

where *x* represents the feature values, *y* is the response (pCR), $h_\theta \left( x \right) = x^T\theta + b$ is the linear function with weight *θ* and bias *b*. N-1 is the number of training samples (1 sample for held out testing), *K* is the number of features, *λ* is the LASSO regularization penalty weight, *α*_1_ and *α*_2_ imposed the class weights. We set the number of maximum iteration = 100, tolerance = 1e-4. The hyper-parameters to be searched is the LASSO regularization penalty weight *λ* from 0.1 to 1.0 with step = 0.1. The class weights *α*_1_ and *α*_2_ were used for balanced learning by adjusting weights inversely proportional to pCR frequencies in the input data.

We then adopted five measurements to evaluate the results, namely, AUC (area under the ROC curve), F1 score, precision (positive predictive value), recall, and negative predictive value. AUC was evaluated in scikit-learn (version 0.23.2). F1 score, precision, and recall were evaluated in scikit-learn with “macro” average method. A well-discriminated model would have an AUC close to 1. We considered an AUC > 0.85 being a well-performed prediction, and an AUC > 0.75 being an adequate prediction. Precision is the fraction of true positive classification among the positive results classified by algorithm and reflects how likely it is that a patient will have pCR. The higher precision indicates an algorithm’s result is reliable. Recall is the fraction of true positive classification among all the true samples and shows the ability of the model to correctly identify the patients with pCR. Note that during this calculation, “positive” stands for a pCR. The F1 score is formulated as

$$F1 = \frac{{2\left( {precision \times recall} \right)}}{{precision + recall}},$$

which is the harmonic mean of the precision and recall, reflecting the learning accuracy. To compare the performances between IMPRESS features and pathologists’ assessed features (both include clinical features), LASSO-regularized logistic regression was used for both features. The model and training schemes as well as evaluation metrics were remained same as before.

### Statistical analyses

We compared the distributions of IMPRESS and clinical features between HER2+ and TNBC cohorts using Mann–Whitney *U* test. The fold change was calculated by the ratio of the median feature values between HER2+ and TNBC cohorts. Student’s *t*-test was adopted for comparing pair-wised AUCs from different trials. Spearman’s rank correlation coefficients was adopted for calculating the relationships between features and pCR, the relationships among IMPRESS features, and the relationships between IMPRESS features and residual tumor sizes. It provides a correlation coefficient *ρ* and a *P*-value. All *P*-values were two-sided, followed with B&H procedure for multiple test adjustment (FDR = 0.05); Adjusted *P*-values < 0.05 were deemed statistically significant.

### Reporting summary

Further information on research design is available in the Nature Research Reporting Summary linked to this article.

## Supplementary information

Supplementary Material

REPORTING SUMMARY

## Data Availability

IMPRESS data and features extracted from H&E-stained and IHC-stained whole-slide images are available at https://tinyurl.com/IMPRESS-DATA. The annotated breast cancer slides used for training H&E image segmentation model are available from Amgad et al.^39^ dataset (https://goo.gl/cNM4EL).

## Code availability

The entire pipeline is available from GitHub at https://github.com/huangzhii/IMPRESS. Detailed software versions are: OpenSlide (version 1.1.2), PyTorch (version 1.6.0), torchvision (version 0.7.0), scikit-learn (version 0.23.2), pillow (version 7.2.0), OpenCV (version 4.4.0), and pandas (version 1.0.5).
