# Supplementary material for: Artificial intelligence reveals features associated with breast cancer neoadjuvant chemotherapy responses from multi-stain histopathologic images
Source: NPJ Precis Oncol. 2023 Jan 27;7:14. doi: 10.1038/s41698-023-00352-5 (PMC9883475; doi:10.1038/s41698-023-00352-5)
Supplement: Supplementary file 1 — Supplementary Material [file 41698_2023_352_MOESM1_ESM.pdf]

Supplementary Information

Artificial Intelligence Reveals Features Associated with Breast Cancer Neoadjuvant Chemotherapy Responses from Multi-stain Histopathologic Images

Zhi Huang[1,2,†], Wei Shao[3,†], Zhi Han[3,4,11,†], Ahmad Mahmoud Alkashash[5], Carlo De la Sancha[5], Anil V. Parwani[6], Hiroaki Nitta[7], Yanjun Hou[8], Tongxin Wang[9], Paul Salama[2], Maher Rizkalla[2], Jie Zhang[10], Kun Huang[2,4,11,*], and Zaibo Li[6,*]

[1] School of Electrical and Computer Engineering, Purdue University, West Lafayette, IN, 47907, USA
[2] Department of Electrical and Computer Engineering, Indiana University – Purdue University Indianapolis, Indianapolis, IN, 46202, USA
[3] Department of Medicine, Indiana University School of Medicine, Indianapolis, IN, 46202, USA
[4] Regenstrief Institute, Indianapolis, IN, 46202, USA
[5] Department of Pathology, Indiana University School of Medicine, Indianapolis, IN, 46202, USA
[6] Department of Pathology, The Ohio State University Wexner Medical Center, Columbus, OH, 43210, USA
[7] Roche Tissue Diagnostics, 1910 E. Innovation Park Drive, Tucson, AZ, 85755, USA
[8] University Hospitals Cleveland Medical Center, Case Western Reserve University, 11100 Euclid Avenue, Cleveland, OH, 44106, USA
[9] Department of Computer Science, Indiana University Bloomington, Bloomington, IN, 47408, USA
[10] Department of Medical and Molecular Genetics, Indiana University School of Medicine, Indianapolis, IN, 46202, USA
[11] Department of Biostatistics and Health Data Science, Indiana University School of Medicine, Indianapolis, IN, 46202, USA

[†] These authors contributed equally to this work.
* To whom the correspondence should be addressed: Zaibo Li (Zaibo.Li@osumc.edu) and Kun Huang (kunhuang@iu.edu).

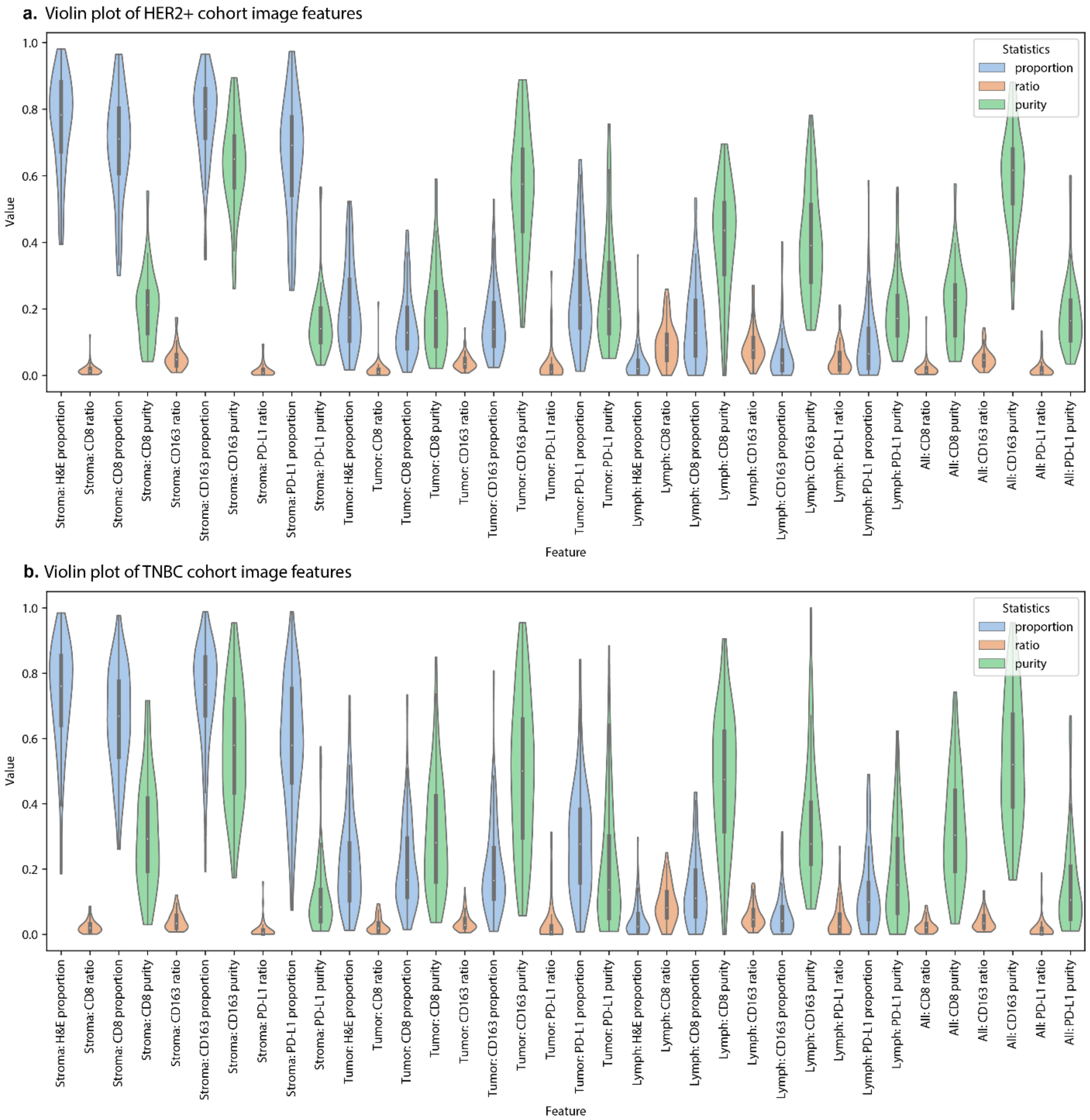

Supplementary Figure 1. Violin plot of IMPRESS feature expressions in HER2+ cohort (A) and TNBC cohort (B). For boxplot inside the violin plot, the interior white dot represents the median value, the upper and lower box edges represent 75th and 25th percentile, and the upper and lower bars represent the 90th and 10th percentiles, respectively.

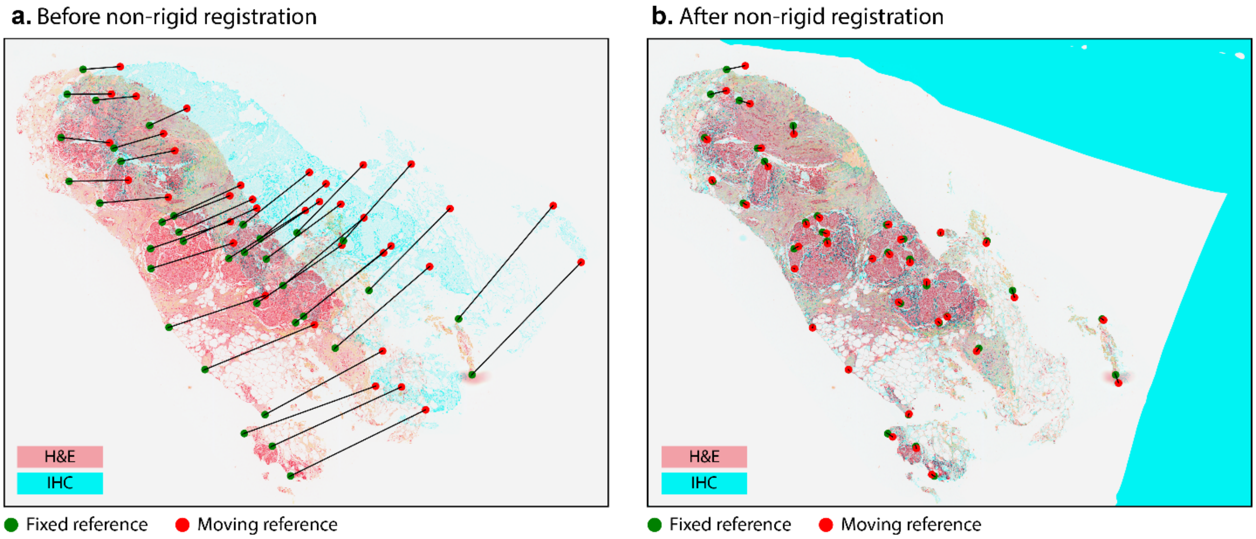

Supplementary Figure 2. An example H&E tissue (fixed reference) and the corresponding IHC tissue (moving reference) before the non-rigid registration (A) and after the non-rigid registration (B). Figure best viewed in color.

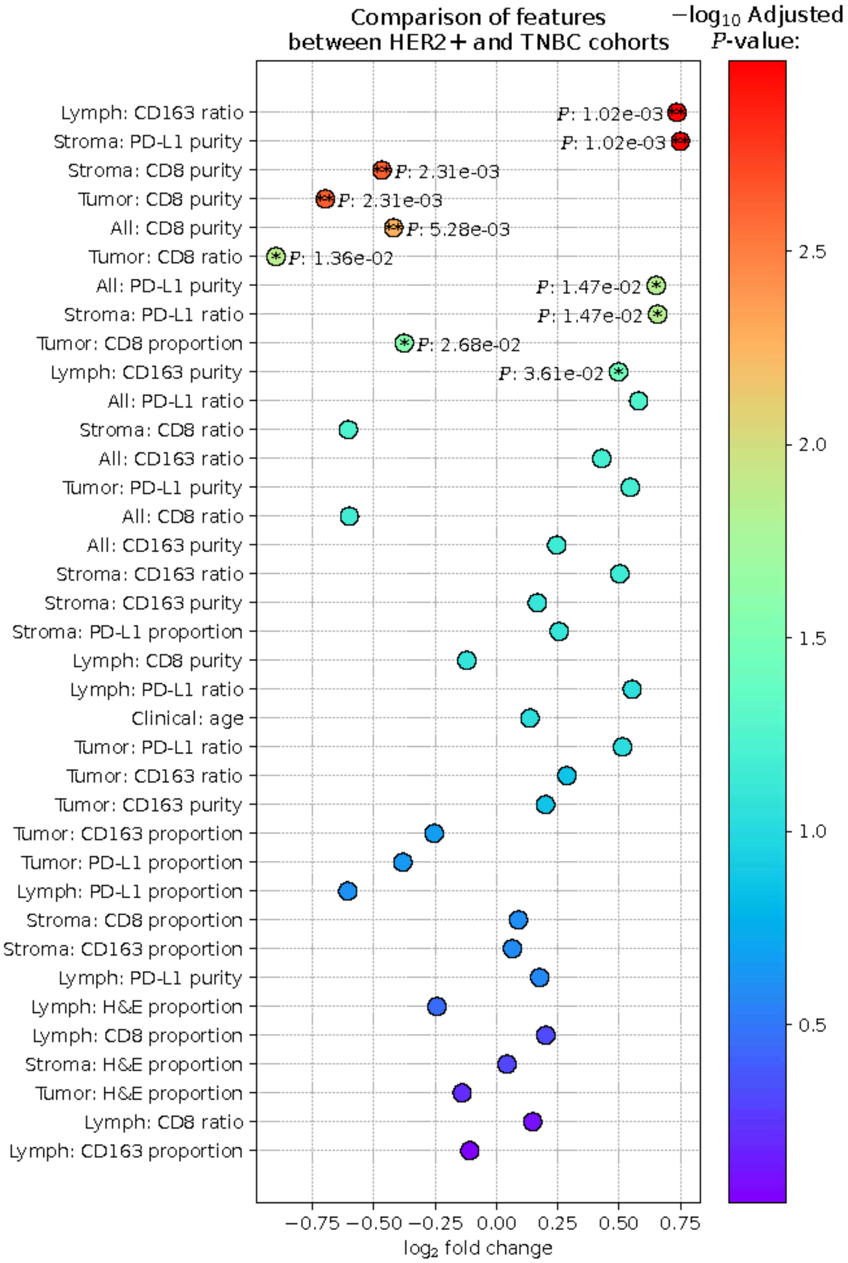

Supplementary Figure 3. Extracted IMPRESS and clinical features comparison between HER2+ and TNBC cohorts. Two-sided P-values were calculated based on Mann–Whitney U test, followed with B&H procedure for multiple test adjustment (FDR = 0.05). The fold change was calculated by the ratio of the median feature values between HER2+ and TNBC cohorts. Figure best viewed in color. Symbol for adjusted P-values: *: < 0.05, **: < 0.01, ***: < 0.001.

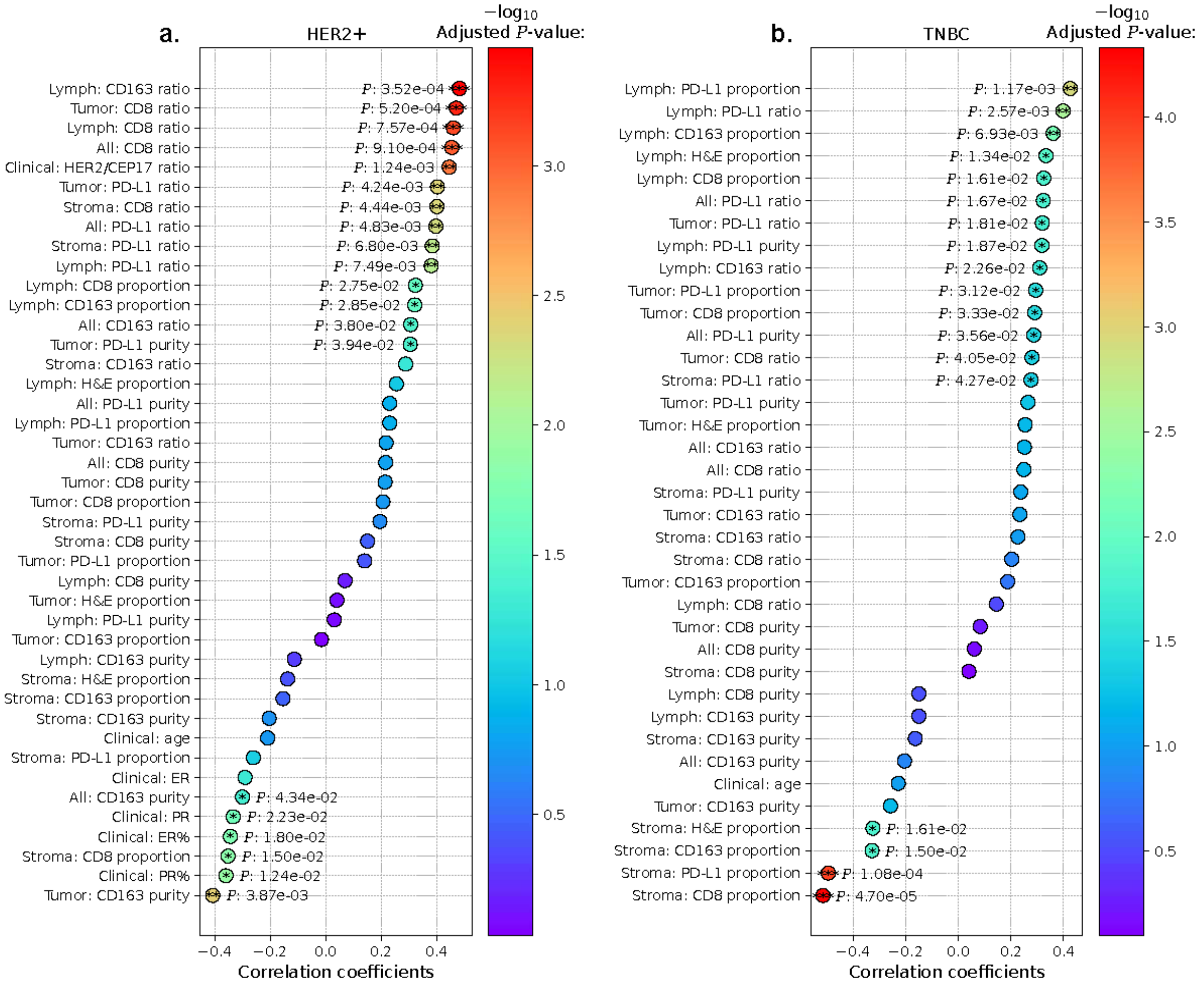

Supplementary Figure 4. (A) Univariate feature analysis with pCR in HER2+ cohort. (B) Univariate feature analysis with pCR in TNBC cohort. Two-sided P-values were calculated based on Spearman's rank correlations, followed with B&H procedure for multiple test adjustment (FDR = 0.05). Figure best viewed in color. Symbol for adjusted P-values: *: < 0.05, **: < 0.01, ***: < 0.001.

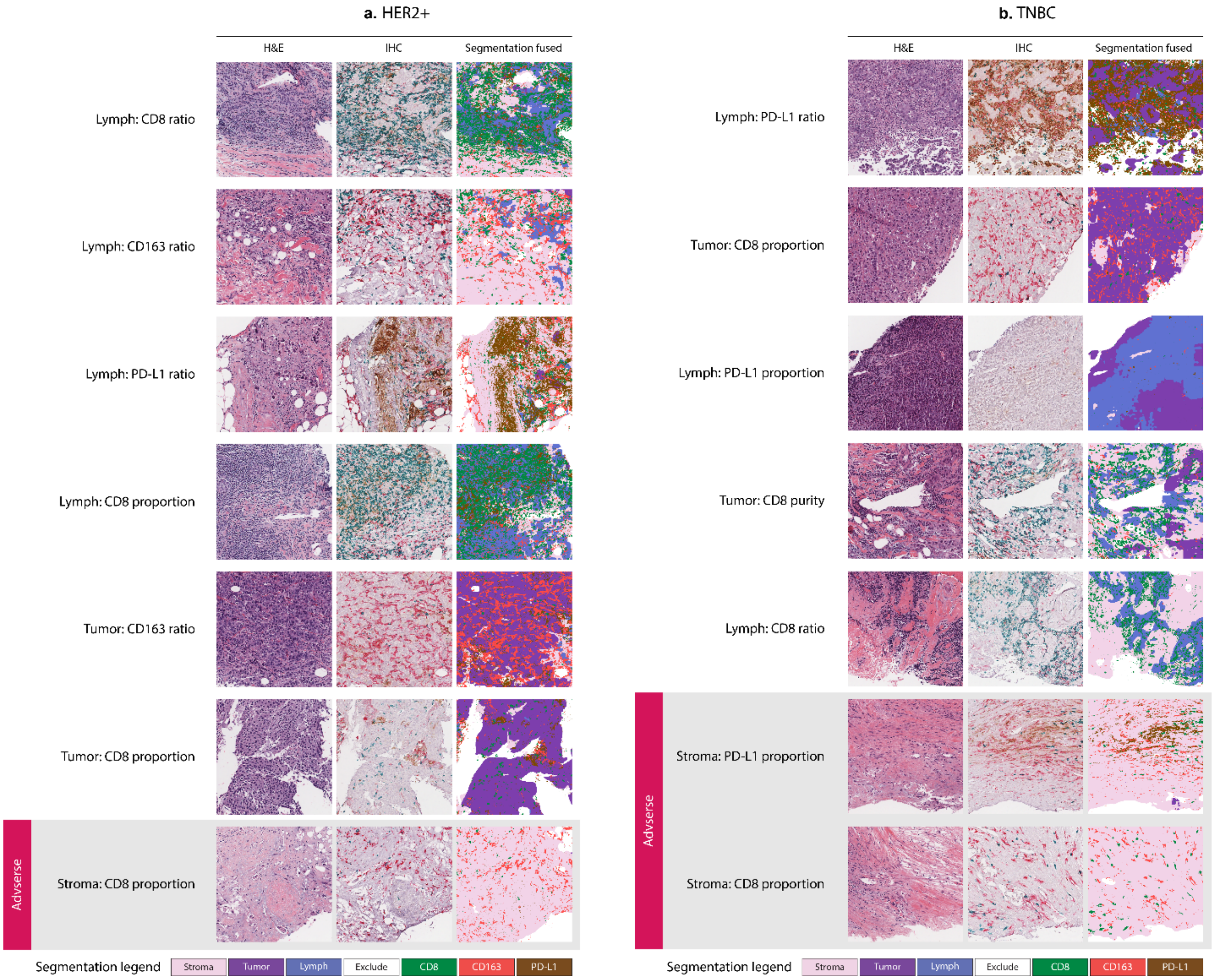

Supplementary Figure 5. Selected representative patches in HER2+ cohort (A) and TNBC cohort (B). Patches were derived from patient WSIs which achieved highest IMPRESS feature values among cohorts. Adverse prognostic markers were highlighted. Figure best viewed in color.

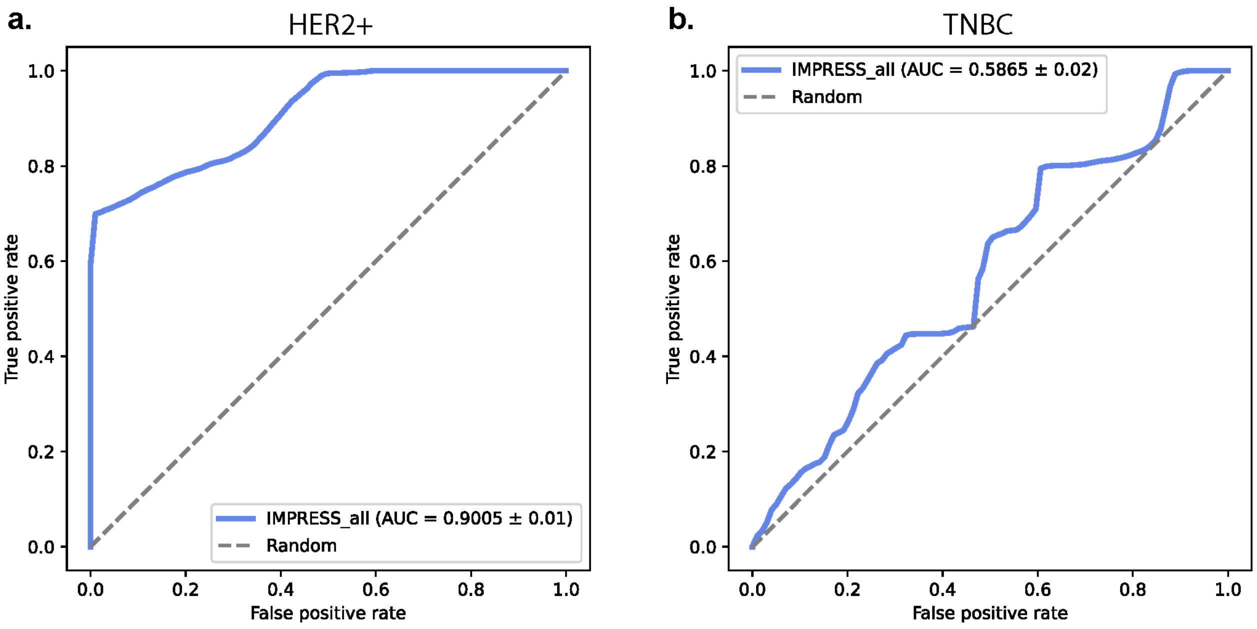

Supplementary Figure 6. Receiver operating characteristic curves for external validation dataset. (A) HER2+ cohort; (B) TNBC cohort.

Supplementary Table 1. List of 36 IMPRESS features constructed from H&E and IHC images. 3 features can be constructed only from H&E image, 3 features can be only constructed from IHC image.

	Feature name	Explanation	Data source
1	*Stroma: CD8 ratio*	The area ratio of CD8 to stromal region.	H&E+IHC
2	*Stroma: CD163 ratio*	The area ratio of CD163 to stromal region.	H&E+IHC
3	*Stroma: PD-L1 ratio*	The area ratio of PD-L1 to stromal region.	H&E+IHC
4	*Stroma: CD8 proportion*	The area ratio of CD8 in stromal region to all H&E regions.	H&E+IHC
5	*Stroma: CD163 proportion*	The area ratio of CD163 in stromal region to all H&E regions.	H&E+IHC
6	*Stroma: PD-L1 proportion*	The area ratio of PD-L1 in stromal region to all H&E regions.	H&E+IHC
7	*Stroma: CD8 purity*	In stromal region, the area ratio of CD8 to all IHC markers.	H&E+IHC
8	*Stroma: CD163 purity*	In stromal region, the area ratio of CD163 to all IHC markers.	H&E+IHC
9	*Stroma: PD-L1 purity*	In stromal region, the area ratio of PD-L1 to all IHC markers.	H&E+IHC
10	*Tumor: CD8 ratio*	The area ratio of CD8 to tumoral region.	H&E+IHC
11	*Tumor: CD163 ratio*	The area ratio of CD163 to tumoral region.	H&E+IHC
12	*Tumor: PD-L1 ratio*	The area ratio of PD-L1 to tumoral region.	H&E+IHC
13	*Tumor: CD8 proportion*	The area ratio of CD8 in tumoral region to all H&E regions.	H&E+IHC
14	*Tumor: CD163 proportion*	The area ratio of CD163 in tumoral region to all H&E regions.	H&E+IHC
15	*Tumor: PD-L1 proportion*	The area ratio of PD-L1 in tumoral region to all H&E regions.	H&E+IHC
16	*Tumor: CD8 purity*	In tumoral region, the area ratio of CD8 to all IHC markers.	H&E+IHC
17	*Tumor: CD163 purity*	In tumoral region, the area ratio of CD163 to all IHC markers.	H&E+IHC
18	*Tumor: PD-L1 purity*	In tumoral region, the area ratio of PD-L1 to all IHC markers.	H&E+IHC
19	*Lymph: CD8 ratio*	The area ratio of CD8 to lymphocytes aggregated region.	H&E+IHC
20	*Lymph: CD163 ratio*	The area ratio of CD163 to lymphocytes aggregated region.	H&E+IHC
21	*Lymph: PD-L1 ratio*	The area ratio of PD-L1 to lymphocytes aggregated region.	H&E+IHC
22	*Lymph: CD8 proportion*	The area ratio of CD8 in lymphocytes aggregated region to all H&E regions.	H&E+IHC
23	*Lymph: CD163 proportion*	The area ratio of CD163 in lymphocytes aggregated region to all H&E regions.	H&E+IHC
24	*Lymph: PD-L1 proportion*	The area ratio of PD-L1 in lymphocytes aggregated region to all H&E regions.	H&E+IHC
25	*Lymph: CD8 purity*	In lymphocytes aggregated region, the area ratio of CD8 to all IHC markers.	H&E+IHC
26	*Lymph: CD163 purity*	In lymphocytes aggregated region, the area ratio of CD163 to all IHC markers.	H&E+IHC
27	*Lymph: PD-L1 purity*	In lymphocytes aggregated region, the area ratio of PD-L1 to all IHC markers.	H&E+IHC
28	*All: CD8 ratio*	The area ratio of CD8 to all H&E regions.	H&E+IHC
29	*All: CD163 ratio*	The area ratio of CD163 to all H&E regions.	H&E+IHC
30	*All: PD-L1 ratio*	The area ratio of PD-L1 to all H&E regions.	H&E+IHC
31	*All: CD8 purity*	In all H&E regions, the area ratio of CD8 to all IHC markers.	IHC
32	*All: CD163 purity*	In all H&E regions, the area ratio of CD163 to all IHC markers.	IHC
33	*All: PD-L1 purity*	In all H&E regions, the area ratio of PD-L1 to all IHC markers.	IHC
34	*Stroma: H&E proportion*	The area ratio of stromal region to all H&E regions.	H&E
35	*Tumor: H&E proportion*	The area ratio of tumoral region to all H&E regions.	H&E
36	*Lymph: H&E proportion*	The area ratio of lymphocytes aggregated region to all H&E regions.	H&E

Supplementary Table 2. Confusion matrix in H&E segmentation results for HER2+ (A) and TNBC (B). Exclude: excluded region; Stroma: stromal region; Tumor: tumoral region; Lymph: lymphocytes aggregated region.

(A) HER2+

		Label predicted			
		**Exclude**	**Stroma**	**Tumor**	**Lymph**
**Label truth**	**Exclude**	1013128	246813	20645	196
	**Stroma**	51214	3966505	52017	8137
	**Tumor**	43656	195743	853359	2008
	**Lymph**	11	32339	7855	59974

(B) TNBC

		Label predicted			
		**Exclude**	**Stroma**	**Tumor**	**Lymph**
**Label truth**	**Exclude**	1204848	140546	73137	375
	**Stroma**	113180	3596604	175049	2014
	**Tumor**	16424	213614	822769	17049
	**Lymph**	0	32729	31204	114058

Supplementary Table 3. Confusion matrix in IHC segmentation results for HER2+ (A) and TNBC (B). Exclude: excluded background region; CD8: CD8 region; CD163: CD163 region; PD-L1: PD-L1 region.

(A) HER2+

		Label predicted			
		**Exclude**	**Stroma**	**Tumor**	**Lymph**
**Label truth**	**Exclude**	980285	8799	17195	16166
	**Stroma**	15168	83190	120	2785
	**Tumor**	19796	111	54759	1068
	**Lymph**	24832	4186	607	81653

(B) TNBC

		Label predicted			
		**Exclude**	**Stroma**	**Tumor**	**Lymph**
**Label truth**	**Exclude**	1067720	4508	6006	9970
	**Stroma**	11948	59050	37	1520
	**Tumor**	16463	50	49179	7365
	**Lymph**	18702	1037	2867	54298

Supplementary Table 4. Non-rigid registration performances of HER2+ and TNBC cohorts.

		Before registration		After registration	
**Cohort**	**Metric**	**Mean**	**Median**	**Mean**	**Median**
**HER2+**	distance (*μm*)	374.01	278.73	33.31	18.23
	median *rTRE*	6.04	5.35	0.59	0.40
**TNBC**	distance (*μm*)	627.66	482.14	47.78	27.13
	median *rTRE*	6.46	5.85	0.44	0.27

Supplementary Table 5. Feature importance in HER2+ and TNBC cohorts. Experiments are repeated 20 times with different random seeds in leave-one-out cross-validation setting. Top 5 favorable and adverse prognosis marker for HER2+ and TNBC cohorts are reported respectively. Values are reported in mean ± standard deviation.

	Favorable prognostic markers				Adverse prognostic markers			
**Cohort**	**Rank**	**H&E region**	**Feature**	**Coefficients**	**Rank**	**H&E region**	**Feature**	**Coefficients**
**HER2+**	1	Lymph	CD8 ratio	0.7879 ± 0.0992	1	Clinical	age	−0.8638 ± 0.1346
	2	Lymph	CD163 ratio	0.7141 ± 0.0770	2	Stroma	CD8 proportion	−0.4627 ± 0.0352
	3	Clinical	HER2/CEP17 ratio	0.6414 ± 0.1010	3	Clinical	ER%	−0.4429 ± 0.0536
	4	Lymph	PD-L1 ratio	0.3748 ± 0.0653	4	Clinical	PR+/−	−0.4158 ± 0.0818
	5	Tumor	CD163 ratio	0.2219 ± 0.0726	5	Clinical	PR%	−0.2396 ± 0.0568
**TNBC**	1	Lymph	PD-L1 ratio	0.4412 ± 0.2300	1	Stroma	CD8 proportion	−0.5878 ± 0.1024
	2	Lymph	PD-L1 proportion	0.2409 ± 0.1095	2	Clinical	age	−0.0608 ± 0.0762
	3	Tumor	CD8 proportion	0.1249 ± 0.1827	3	Tumor	PD-L1 ratio	−0.0545 ± 0.1084
	4	Tumor	CD8 purity	0.0847 ± 0.1129	4	Stroma	CD8 ratio	−0.0472 ± 0.1059
	5	Lymph	CD163 proportion	0.0452 ± 0.0994	5	Lymph	CD8 purity	−0.0249 ± 0.0739

Supplementary Table 6. Student's t-test results by comparing IMPRESS and clinical features of pCR cases against residual tumor cases. Features are sorted by adjusted P-values in ascending order. Symbol for adjusted P-values: *: < 0.05; **: < 0.01; ***: < 0.001. Significant P-values were highlighted in red.

	HER2+			TNBC		
**Rank**	**Feature names**	**t statistics**	**Adjusted P-value**	**Feature names**	**t statistics**	**Adjusted P-value**
1	*Lymph: CD163 ratio*	3.594455	**0.016305 ***	*Stroma: CD8 proportion*	-4.666563	**0.000623 *****
2	*Lymph: CD8 ratio*	3.541561	**0.016305 ***	*Stroma: PD-L1 proportion*	-4.242431	**0.001389 ****
3	*Clinical: HER2/CEP17 ratio*	3.226637	**0.028426 ***	*Lymph: PD-L1 ratio*	3.517819	**0.008316 ****
4	*Stroma: CD8 proportion*	-3.072326	**0.029197 ***	*Lymph: PD-L1 proportion*	3.488683	**0.008316 ****
5	*Lymph: PD-L1 ratio*	3.042724	**0.029197 ***	*Lymph: CD163 proportion*	3.376036	**0.009426 ****
6	*Tumor: CD163 purity*	-2.971149	**0.029842 ***	*Stroma: CD163 proportion*	-3.076857	**0.019193 ***
7	*Clinical: ER%*	-2.902422	**0.031035 ***	*Lymph: CD8 proportion*	2.948188	**0.023794 ***
8	*Lymph: CD8 proportion*	2.825048	**0.031122 ***	*Stroma: H&E proportion*	-2.830533	**0.028934 ***
9	*Clinical: PR%*	-2.810583	**0.031122 ***	*Lymph: PD-L1 purity*	2.736953	**0.031908 ***
10	*Clinical: PR*	-2.741490	**0.033801 ***	*Tumor: CD8 proportion*	2.712844	**0.031908 ***
11	*Lymph: CD163 proportion*	2.370234	0.069208	*Lymph: H&E proportion*	2.552162	**0.044346 ***
12	*Clinical: ER*	-2.353331	0.069208	*Tumor: PD-L1 proportion*	2.166371	0.098426
13	*All: CD163 ratio*	2.341825	0.069208	*Tumor: H&E proportion*	2.160806	0.098426
14	*Stroma: CD8 ratio*	2.278235	0.069208	*Clinical: age*	-2.055501	0.116414
15	*Tumor: PD-L1 purity*	2.255415	0.069208	*Tumor: CD163 purity*	-1.975628	0.129875
16	*Stroma: CD163 ratio*	2.249187	0.069208	*Stroma: PD-L1 ratio*	1.920414	0.137381
17	*All: CD8 ratio*	2.247796	0.069208	*All: PD-L1 purity*	1.890408	0.137943
18	*All: CD163 purity*	-2.205629	0.069208	*Lymph: CD163 ratio*	1.827774	0.143418
19	*All: PD-L1 ratio*	2.204862	0.069208	*Tumor: PD-L1 purity*	1.819611	0.143418
20	*Clinical: age*	-2.078504	0.088090	*All: PD-L1 ratio*	1.788630	0.145336
21	*Stroma: PD-L1 proportion*	-2.035503	0.088498	*Stroma: PD-L1 purity*	1.695521	0.163010
22	*Stroma: PD-L1 ratio*	2.034231	0.088498	*Tumor: CD163 proportion*	1.685473	0.163010
23	*Tumor: CD8 ratio*	1.770354	0.149279	*All: CD163 purity*	-1.659165	0.164301
24	*Tumor: PD-L1 ratio*	1.665460	0.165989	*Stroma: CD163 ratio*	1.549523	0.194783
25	*Lymph: PD-L1 proportion*	1.661584	0.165989	*All: CD163 ratio*	1.512692	0.198751
26	*Tumor: CD163 ratio*	1.656927	0.165989	*Lymph: CD8 ratio*	1.496247	0.198751
27	*Lymph: H&E proportion*	1.505547	0.213780	*Tumor: CD8 ratio*	1.354115	0.247509
28	*Stroma: CD163 purity*	-1.481817	0.215433	*Stroma: CD163 purity*	-1.161706	0.323018
29	*All: PD-L1 purity*	1.449248	0.220826	*Lymph: CD8 purity*	-1.148333	0.323018
30	*Stroma: CD163 proportion*	-1.328562	0.263135	*Tumor: PD-L1 ratio*	1.132209	0.323018
31	*Stroma: H&E proportion*	-1.312893	0.263135	*All: CD8 ratio*	1.015053	0.374804
32	*All: CD8 purity*	1.286521	0.266704	*Lymph: CD163 purity*	-0.878047	0.443200
33	*Tumor: CD8 proportion*	1.242422	0.278619	*Tumor: CD163 ratio*	0.695981	0.548319
34	*Tumor: CD8 purity*	1.179454	0.300020	*Tumor: CD8 purity*	0.561242	0.617441
35	*Tumor: PD-L1 proportion*	1.156670	0.302390	*Stroma: CD8 ratio*	0.550337	0.617441
36	*Stroma: PD-L1 purity*	1.005040	0.372066	*All: CD8 purity*	0.349839	0.747858

37	*Stroma: CD8 purity*	0.938257	0.399425	*Stroma: CD8 purity*	0.148199	0.882666
38	*Tumor: H&E proportion*	0.830702	0.448729			
39	*Lymph: CD163 purity*	-0.817853	0.448729			
40	*Lymph: CD8 purity*	0.630844	0.557065			
41	*Lymph: PD-L1 purity*	0.246588	0.825729			
42	*Tumor: CD163 proportion*	-0.043845	0.965174			

Supplementary Table 7. Spearman's rank correlation coefficient statistics between IMPRESS features and residual cancer burden (RCB) values in HER2+ and TNBC cohorts. Features are sorted by adjusted P-values in ascending order. Symbol for adjusted P-values: *: < 0.05; **: < 0.01; ***: < 0.001. Significant P-values were highlighted in red.

	HER2+			TNBC		
**Rank**	**Feature names**	**Spearman ρ**	**Adjusted P-value**	**Feature names**	**Spearman ρ**	**Adjusted P-value**
1	*Lymph: CD163 ratio*	-0.501054	**0.001208 ****	*Stroma: CD8 proportion*	0.434782	**0.011902 ***
2	*Lymph: CD8 ratio*	-0.474161	**0.001782 ****	*Stroma: PD-L1 proportion*	0.398219	**0.020133 ***
3	*Tumor: CD8 ratio*	-0.459897	**0.002034 ****	*Lymph: PD-L1 proportion*	-0.380247	**0.023275 ***
4	*All: CD8 ratio*	-0.442734	**0.002828 ****	*Lymph: CD8 proportion*	-0.318356	0.078079
5	*Stroma: CD8 ratio*	-0.381315	**0.013308 ***	*Lymph: CD163 proportion*	-0.313238	0.078079
6	*Tumor: PD-L1 ratio*	-0.374829	**0.013308 ***	*Lymph: H&E proportion*	-0.308882	0.078079
7	*Tumor: CD163 purity*	0.370868	**0.013308 ***	*Lymph: PD-L1 ratio*	-0.298432	0.085415
8	*Lymph: PD-L1 ratio*	-0.369318	**0.013308 ***	*All: PD-L1 ratio*	-0.251490	0.180450
9	*All: PD-L1 ratio*	-0.365530	**0.013308 ***	*All: PD-L1 purity*	-0.244230	0.180450
10	*Stroma: PD-L1 ratio*	-0.363377	**0.013308 ***	*Stroma: H&E proportion*	0.240374	0.180450
11	*Stroma: CD8 proportion*	0.335825	**0.024939 ***	*Tumor: PD-L1 ratio*	-0.237279	0.180450
12	*Lymph: CD8 proportion*	-0.323799	**0.030667 ***	*Lymph: PD-L1 purity*	-0.236280	0.180450
13	*Lymph: CD163 proportion*	-0.320585	**0.030667 ***	*Lymph: CD163 ratio*	-0.224116	0.205650
14	*Tumor: PD-L1 purity*	-0.288612	0.058911	*Stroma: PD-L1 ratio*	-0.220497	0.205650
15	*All: CD163 purity*	0.282930	0.062082	*Tumor: PD-L1 purity*	-0.211619	0.211425
16	*All: CD163 ratio*	-0.273946	0.070192	*Stroma: PD-L1 purity*	-0.208619	0.211425
17	*Stroma: CD163 ratio*	-0.247657	0.110738	*Tumor: CD163 purity*	0.207548	0.211425
18	*Tumor: CD8 purity*	-0.241343	0.117597	*Tumor: CD8 proportion*	-0.203216	0.214561
19	*All: CD8 purity*	-0.237497	0.119504	*Stroma: CD163 proportion*	0.196170	0.217098
20	*Lymph: H&E proportion*	-0.229489	0.130990	*Tumor: PD-L1 proportion*	-0.196003	0.217098
21	*Stroma: PD-L1 proportion*	0.220535	0.145700	*Tumor: H&E proportion*	-0.184291	0.248396
22	*Lymph: PD-L1 proportion*	-0.217550	0.146004	*Tumor: CD8 ratio*	-0.171889	0.285410
23	*All: PD-L1 purity*	-0.215024	0.146004	*All: CD163 purity*	0.167914	0.287296
24	*Stroma: CD163 purity*	0.200301	0.168731	*All: CD8 ratio*	-0.165391	0.287296
25	*Tumor: CD163 ratio*	-0.200014	0.168731	*All: CD163 ratio*	-0.156821	0.310888
26	*Stroma: PD-L1 purity*	-0.198234	0.168731	*Stroma: CD163 ratio*	-0.140111	0.373130
27	*Tumor: CD8 proportion*	-0.196139	0.168731	*Tumor: CD163 ratio*	-0.125543	0.402711
28	*Stroma: CD8 purity*	-0.170194	0.239141	*Stroma: CD8 ratio*	-0.125448	0.402711
29	*Stroma: CD163 proportion*	0.132281	0.379133	*Lymph: CD8 ratio*	-0.125162	0.402711
30	*Tumor: PD-L1 proportion*	-0.115835	0.443951	*Lymph: CD8 purity*	0.120020	0.403368
31	*Lymph: CD163 purity*	0.107283	0.472141	*Stroma: CD163 purity*	0.119401	0.403368
32	*Stroma: H&E proportion*	0.095343	0.518670	*Lymph: CD163 purity*	0.110618	0.432231
33	*Lymph: CD8 purity*	-0.090722	0.527063	*Tumor: CD163 proportion*	-0.094931	0.496983
34	*Tumor: CD163 proportion*	0.022932	0.910140	*Tumor: CD8 purity*	-0.087194	0.522288
35	*Tumor: H&E proportion*	-0.012714	0.948208	*All: CD8 purity*	-0.045561	0.741317
36	*Lymph: PD-L1 purity*	-0.000718	0.995584	*Stroma: CD8 purity*	-0.013259	0.917181

Supplementary Table 8. Performances in HER2+ and TNBC external validation cohorts. Experiments used the previous established LASSO-regularized logistic regression model (repeated 20 times with different random seeds in leave-one-out cross-validation setting). mean value ± standard deviation are reported. PPV: positive predictive value; NPV: negative predictive value.

Cohort	AUC	F1 score	Precision (PPV)	Recall	NPV
**HER2+**	0.9005±0.0060	0.5714±0.0000	1.0000±0.0000	0.4000±0.0000	0.6250±0.0000
**TNBC**	0.5865±0.0157	0.4882±0.0148	0.6286±0.0479	0.4000±0.0000	0.5582±0.0166

Supplementary Table 9. HER2+ study cohort metadata.

Unique ID	Age	HER2/CEP17 ratio	ER (+-1/-0)	ER%	ER (1-3+)	PR (+/-)	PR%	PR (1-3+)	Residual tumor size (cm)	pCR (no-0, yes-1)	RCB value
061	46	4.58	1	95	3	1	95	3	1.4	0	1.375
062	51	6.03	1	98	3	1	90	3	7	0	3.213
063	57	4.79	1	95	2	1	95	2	0.9	0	2.066
064	55	3.09	1	95	3	1	10	2	4.4	0	4.138
065	66	6.34	1	95	3	0	0	0		1	
066	75	4.65	0	0	0	0	0	0	0.8	0	1.131
067	57	3.13	0	0	0	0	0	0	1.4	0	3.046
068	62	3.93	0	0	0	0	0	0	2.1	0	3.745
069	62	7.94	0	0	0	0	0	0		1	
070	65	6.04	1	95	3	1	50	2	0.7	0	0.933
071	51	7.42	1	90	3	1	80	2		1	
072	51	7.06	1	90	3	1	60	2		1	
073	60	13.32	0	0	0	0	0	0		1	
074	56	4.37	1	95	3	0	0	0	0.7	0	1.075
075	64	2.28	0	0	0	0	0	0		1	
076	70	9.32	0	0	0	0	0	0		1	
077	76	6.06	1	95	3	1	95	3	0.3	0	1.141
078	37	6.43	0	0	0	0	0	0		1	
079	56	2.09	0	0	0	0	0	0	2.5	0	2.25
080	56	1.23	0	0	0	0	0	0	2.5	0	2.39
082	64	3.82	1	80	2	0	0	0		1	
083	30	2.30	0	0	0	0	0	0		1	
084	45	5.17	1	1	1	0	0	0		1	
085	56	10.40	1	90	2	0	0	0		1	
086	52	6.75	0	0	0	0	0	0		1	
087	61	6.70	1	20	2	0	0	0		1	
088	30	5.46	0	0	0	0	0	0		1	
089	54	11.20	0	0	0	0	0	0		1	
090	59	3.50	0	0	0	0	0	0	1.3	0	3.084
091	34	7.60	0	0	0	0	0	0		1	
092	55	12.40	1	40	2	0	0	0		1	
093	65	8.15	1	95	3	1	50	2	0.25	0	1.085
094	67	7.13	0	0	0	0	0	0		1	
095	37	7.06	0	0	0	0	0	0		1	
096	58	7.61	1	10	1	0	0	0		1	
097	58	4.97	1	30	2	0	0	0	0.8	0	1.388
098	65	8.67	0	0	0	0	0	0	0.21	1	
099	61	13.49	0	0	0	0	0	0		1	
101	57	14.99	0	0	0	0	0	0	2.2	0	1.048
102	40	6.46	1	90	3	1	40	2	0.8	0	0.911
103	57	10.09	1	90	3	1	2	2		1	
104	41	8.66	1	95	3	1	2	1		1	
105	49	2.80	1	95	3	0	0	0	2.1	0	1.387
106	67	8.51	1	90	3	1	9	2	0.6	0	1.102
107	57	8.15	0	0	0	0	0	0		1	
108	54	19.06	0	0	0	0	0	0		1	
109	45	15.00	0	0	0	0	0	0		1	
110	49	2.10	1	85	2	1	95	3	0.5	0	1.472
111	65	1.89	1	50	2	0	0	0	0.3	0	1.141
112	59	2.29	1	100	3	1	30	2		1	
113	48	3.38	0	0	0	0	0	0		1	
114	55	11.78	0	0	0	0	0	0		1	
115	49	7.40	0	0	0	0	0	0	0.6	0	1.812
116	45	22.98	0	0	0	0	0	0		1	
117	59	7.96	0	0	0	0	0	0		1	
118	55	3.59	1	90	3	1	2	2	0.1	0	0.947
119	61	8.73	1	100	3	1	60	3	1.6	0	1.741
120	59	7.61	0	0	0	0	0	0		1	
121	53	6.38	0	0	0	0	0	0		1	
122	48	4.24	1	95	3	1	30	3		1	
123	45	7.66	1	95	3	1	80	2		1	
124	38	7.02	0	0	0	0	0	0		1	

Supplementary Table 10. TNBC study cohort metadata.

Unique ID	Age	Residual tumor size (cm)	pCR (no-0, yes-1)	RCB value
901	35		1	
902	62	0.2	0	2.74
904	50	0.6	0	1.046
905	49	3.4	0	2.313
906	53	1.9	0	3.653
907	74	0.4	0	0.953
908	47	1.2	0	2.421
909	55	1.5	0	0.798
910	57	1.4	0	2.575
911	73		1	
912	46	1.7	0	2.067
913	46	1.8	0	2.014
914	50	0.5	0	1.244
915	66		1	
916	59	0.4	0	1.668
917	46	3.2	0	4.267
918	37		1	
919	53		1	
920	49	0.6	0	2.771
921	46	4.0 (largest span, largest continuous 0.2)	0	2.661
922	40	0.13	0	1.037
923	36		1	
924	55		1	
925	49		1	
926	37	1	0	1.32
927	29		1	
928	57		1	
929	29		1	
931	35	0.02	0	1.532
932	66	0.3	0	1.444
933	59	tumor bed 3.5 (foci 0.1-0.2)	0	3.135
934	31	0.6	0	2.227
935	51	3.9	0	4.031
936	57	0.12	0	2.287
937	52	0.11	0	1.074
938	42		1	
939	39		1	
940	35		1	
941	63	0.7	0	1.156
942	51		1	
943	53		1	
944	66	0.2	0	0.947
945	33		1	
946	65	0.07	0	0.891
947	64		1	
948	54	1.5	0	1.753
949	56	1.1	0	2.843
950	48	1.8	0	1.78
951	56	3.6	0	4.09
952	51	4.9	0	2.51
953	53		1	
954	42		1	
955	53	0.15	0	0.98
956	59	0.53	0	2.308
957	59		1	
958	66	0.3	0	1.444
959	26		1	
960	48		1	
961	48		1	
987	40		1	
988	49	0.1	0	0.841
989	64		1	
990	66		1	
991	66	2.5	0	2.032
