# Supplementary material for: Artificial intelligence reveals features associated with breast cancer neoadjuvant chemotherapy responses from multi-stain histopathologic images
Source: NPJ Precis Oncol. 2023 Jan 27;7:14. doi: 10.1038/s41698-023-00352-5 (PMC9883475; doi:10.1038/s41698-023-00352-5)
Supplement: Supplementary file 2 — REPORTING SUMMARY [file 41698_2023_352_MOESM2_ESM.pdf]

nature portfolio

Corresponding author(s): Zaibo Li

Last updated by author(s): 10-24-2022

Reporting Summary

Nature Portfolio wishes to improve the reproducibility of the work that we publish. This form provides structure for consistency and transparency in reporting. For further information on Nature Portfolio policies, see our Editorial Policies and the Editorial Policy Checklist.

Statistics

For all statistical analyses, confirm that the following items are present in the figure legend, table legend, main text, or Methods section.

n/a	Confirmed	
☐	☒	The exact sample size (*n*) for each experimental group/condition, given as a discrete number and unit of measurement
☐	☒	A statement on whether measurements were taken from distinct samples or whether the same sample was measured repeatedly
☐	☒	The statistical test(s) used AND whether they are one- or two-sided<br>*Only common tests should be described solely by name; describe more complex techniques in the Methods section.*
☐	☒	A description of all covariates tested
☐	☒	A description of any assumptions or corrections, such as tests of normality and adjustment for multiple comparisons
☐	☒	A full description of the statistical parameters including central tendency (e.g. means) or other basic estimates (e.g. regression coefficient) AND variation (e.g. standard deviation) or associated estimates of uncertainty (e.g. confidence intervals)
☐	☒	For null hypothesis testing, the test statistic (e.g. *F*, *t*, *r*) with confidence intervals, effect sizes, degrees of freedom and *P* value noted<br>*Give P values as exact values whenever suitable.*
☒	☐	For Bayesian analysis, information on the choice of priors and Markov chain Monte Carlo settings
☒	☐	For hierarchical and complex designs, identification of the appropriate level for tests and full reporting of outcomes
☒	☐	Estimates of effect sizes (e.g. Cohen's *d*, Pearson's *r*), indicating how they were calculated

Our web collection on statistics for biologists contains articles on many of the points above.

Software and code

Policy information about availability of computer code

Data collection	This study included 62 HER2-positive breast cancer (HER2+) patients and 64 triple-negative breast cancer (TNBC) retrospective patients treated with neoadjuvant chemotherapy (NAC) and follow-up surgical excision. In accord with STARD-2015 guideline, patients with histopathologically confirmed invasive breast carcinoma who underwent NAC from January 2011 to December 2016, those who had underwent surgery after completing NAC were included. HER2 status was determined on biopsy specimens using HER2 IHC and/or fluorescence in situ hybridization (FISH) in accordance with the criteria of American Society of Clinical Oncology (ASCO)/College of American Pathologist (CAP) guidelines updated guidelines. In addition, two sets of external validation datasets were further used to evaluate the developed machine learning model (20 for HER2+, 20 for TNBC, each with 10 pCR cases, 10 residual tumor cases).
Data analysis	We compared the distributions of IMPRESS and clinical features between HER2+ and TNBC cohorts using Mann–Whitney U test. The fold change was calculated by the ratio of the median feature values between HER2+ and TNBC cohorts. Student's t-test was adopted for comparing pair-wised AUCs from different trials. Spearman's rank correlation coefficients was adopted for calculating the relationships between features and pCR, the relationships among IMPRESS features, and the relationships between IMPRESS features and residual tumor sizes. It provides a correlation coefficient ρ and a P-value. All P-values were two-sided, followed with B&H procedure for multiple test adjustment (FDR = 0.05); Adjusted P-values < 0.05 were deemed statistically significant.

For manuscripts utilizing custom algorithms or software that are central to the research but not yet described in published literature, software must be made available to editors and reviewers. We strongly encourage code deposition in a community repository (e.g. GitHub). See the Nature Portfolio guidelines for submitting code & software for further information.

Data

Policy information about availability of data

All manuscripts must include a data availability statement. This statement should provide the following information, where applicable:

- Accession codes, unique identifiers, or web links for publicly available datasets
- A description of any restrictions on data availability
- For clinical datasets or third party data, please ensure that the statement adheres to our policy

The entire pipeline, IMPRESS data, and features extracted from H&E-stained and IHC-stained whole-slide images are available from GitHub at (https://github.com/huangzhii/IMPRESS).

Human research participants

Policy information about studies involving human research participants and Sex and Gender in Research.

Reporting on sex and gender	See Tables in manuscript.
Population characteristics	See Tables in manuscript.
Recruitment	This study included 62 HER2-positive breast cancer (HER2+) patients and 64 triple-negative breast cancer (TNBC) retrospective patients treated with neoadjuvant chemotherapy (NAC) and follow-up surgical excision.
Ethics oversight	This study was approved by the Ohio State University Institutional Research Board. Informed consent was obtained from all individual patients included in the study.

Note that full information on the approval of the study protocol must also be provided in the manuscript.

Field-specific reporting

Please select the one below that is the best fit for your research. If you are not sure, read the appropriate sections before making your selection.

☒ Life sciences ☐ Behavioural & social sciences ☐ Ecological, evolutionary & environmental sciences

For a reference copy of the document with all sections, see nature.com/documents/nr-reporting-summary-flat.pdf

Life sciences study design

All studies must disclose on these points even when the disclosure is negative.

Sample size	N/A
Data exclusions	N/A
Replication	The developed image feature and algorithms were further externally validated from independent cohorts.
Randomization	Experiments are repeated 20 times with different random seeds in leave-one-out cross-validation setting.
Blinding	N/A

Reporting for specific materials, systems and methods

We require information from authors about some types of materials, experimental systems and methods used in many studies. Here, indicate whether each material, system or method listed is relevant to your study. If you are not sure if a list item applies to your research, read the appropriate section before selecting a response.

Materials & experimental systems

n/a	Involved in the study
☒	☐ Antibodies
☒	☐ Eukaryotic cell lines
☒	☐ Palaeontology and archaeology
☒	☐ Animals and other organisms
☒	☐ Clinical data
☒	☐ Dual use research of concern

Methods

n/a	Involved in the study
☒	☐ ChIP-seq
☒	☐ Flow cytometry
☒	☐ MRI-based neuroimaging
